# Overview of the chemistry and biological activities of natural atisine-type diterpenoid alkaloids†

**DOI:** 10.1039/d4ra03305a

**Published:** 2024-07-22

**Authors:** Jiaqi Zheng, Hongjun Jiang, Yuanfeng Yan, Tianpeng Yin

**Affiliations:** a School of Bioengineering, Zunyi Medical University 519041 Zhuhai China ytp@zmu.edu.cn

## Abstract

Atisine-type C_20_-diterpenoid alkaloids (DAs) are a very important class of diterpenoid alkaloids, which play an important role in the biosynthesis of DAs. To date, 87 atisine-type DAs and 11 bis-DAs containing an atisine unit have been reported from five genera in two families. The genus *Spiraea* in Rosaceae family could be regarded as the richest resource for atisine-type DAs, followed by the genera *Delphinium* and *Aconitum* in the Ranunculaceae family. Among the reported atisine-type DAs, several possess unprecedented skeletons. Natural atisine-type DAs have a wide range of biological activities, including antitumor, antiplatelet aggregation, biological control, and anti-inflammatory, analgesic, antiarrhythmic, and cholinesterase inhibitory effects, which are closely related to their structures. In particular, the antiparasitic effect of atisine-type DAs is more prominent than that of other types of DAs, which highlights their potential in antiparasite drug discovery. In summary, the high chemical and biological diversity of atisine-type DAs indicates their great potential as a vast resource for drug discovery.

## Abstract 2

Atisine-type C_20_-diterpenoid alkaloids are a very important class of diterpenoid alkaloids, which possess high chemical and biological diversity.

## Graphical abstract

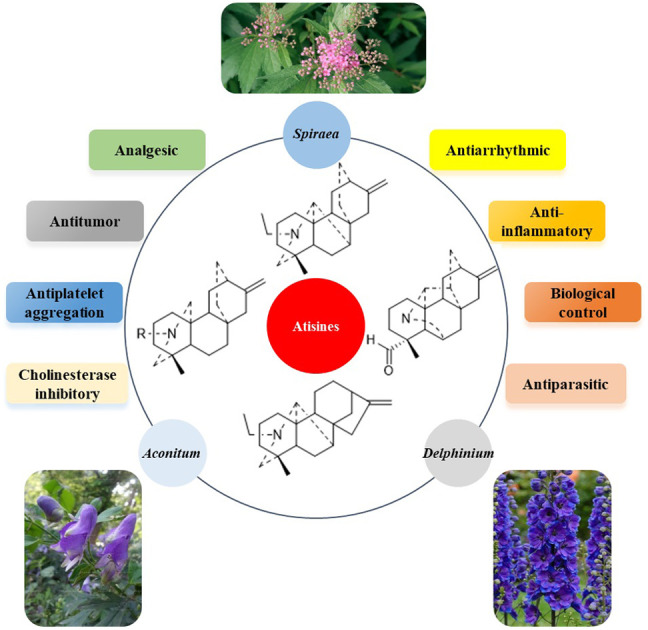

## 1. Introduction

Diterpenoid alkaloids (DAs) are a class of polycyclic nitrogen-containing natural products with complex structures that are formed by the amination of tetracyclic diterpenes or pentacyclic diterpenoids. There are more than 1500 natural DAs, primarily from plants of the genera *Aconitum* and *Delphinium* in the Ranunculaceae family and the genus *Spiraea* in the Rosaceae family.^1,2^ Some representative plants of these genera, such as *A. carmichaelii* and *D. staphisagra*, have a particularly long history of medicinal use in traditional medicines worldwide, and DAs are recognized as their characteristic active ingredients.^3–5^ DAs have various pharmacological effects, especially prominent anti-inflammatory, analgesic, and anti-arrhythmic effects.^6^ Drugs developed from DAs include the analgesics lappaconitine, 3-acetylaconitine, and crassicauline A and the antiarrhythmic drug guanfu-base A.^7,8^ DAs possess complex polycyclic cage-like frameworks decorated with a variety of functional groups, which have attracted the sustained attention of chemists because of their complex and interesting chemical structures.^9^ DAs are primarily divided into four types according to the number of carbons constituting the molecular skeleton and biosynthetic pathways: C_18_-, C_19_-, C_20_-, and bis-DAs. These four categories are further divided into several or dozens of subcategories, and DAs with unprecedented skeletons are still being discovered. C_20_-DAs are second only to C_19_-DAs in terms of the number of compounds, but their chemical structures are more varied, with 9 major categories and 38 types of skeletons reported.^10^

Among C_20_-DAs, atisine-type DAs are a very important class of alkaloids and generally considered biosynthetic precursors of other types of DAs.^11^ Since the discovery of the first compound, atisine, from *A. heterophyllum* (atis in Spanish) in 1896,^12^ approximately 87 atisine-type DAs have been reported. Eleven bis-DAs formed by two atisine molecules or one atisine with one other DA have also been reported in recent decades. Natural atisines exhibit significant physiological effects, including antitumor, antiplatelet aggregation, biological control, anti-inflammatory, analgesic and antiarrhythmic effect, and cholinesterase inhibition effects. The high chemical and biological diversity of atisine-type DAs revealed their great potential in drug discovery.

A series of review papers and monographs on the chemical and pharmacological activities of DAs have been published, some of which include atisine-type DAs.^3,10,13^ However, these studies primarily focused on research involving all types of DAs, and only a small portion of the related research has been devoted to atisine-type DAs. A review on hetisine-type DAs, a group of DAs closely related to atisine-type DAs, has also been published by our group, which has highlighted their prominent antiarrhythmic effects. Previous studies have shown that atisine-type DAs exhibit different biological activities than hetisine-type DAs or other types of DAs, for example, they have outstanding antiparasitic effects. However, no individual or systematic reviews of atisine-type DAs are currently available. There have been a series of studies on atisine-type DAs in the past few decades, and significant progress has been made. Thus, the present review summarizes the research progress on the structural features and biological activities of natural atisine-type DAs to provide a complete overview of the existing knowledge of atisine-type DAs and promote further research and exploitation of atisine-type DAs and the utilization of related medicinal plants.

## 2. Biosynthesis

**Fig. 1:**
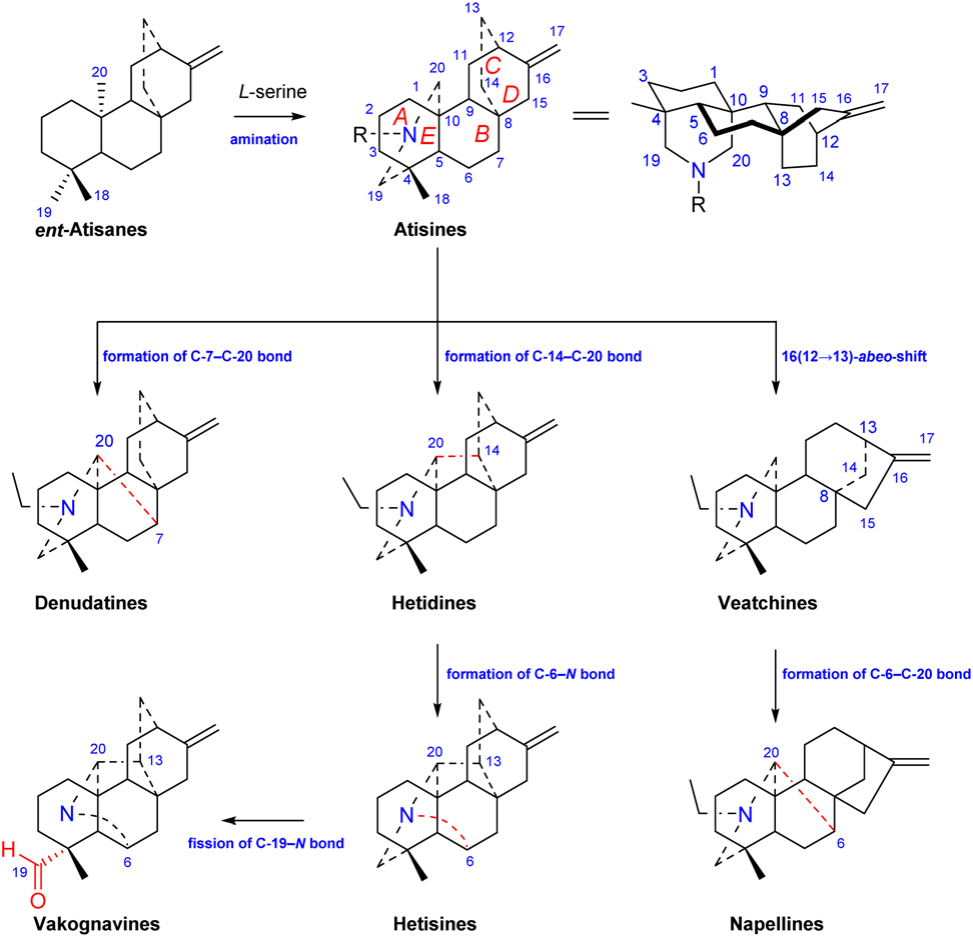
The structures of atisine-type DA and its closely related DAs.

Atisine-type DAs are formed by the amination of *ent*-atisane-type tetracyclic diterpenoids (Fig. 1). The use of *l**-serine* as a main nitrogen source has been supported by Hao *et al.*^14^*Ent*-atisane diterpenoids are generally biosynthesized from isopentenyl pyrophosphate (IPP), and IPP may be coproduced by the MEP (methylerythritol phosphate) and MVA (mevalonic acid) pathways.^15,16^ Briefly, the formation of geranylgeranyl pyrophosphate (GGPP) from three IPP units is catalyzed by geranylgeranyl pyrophosphate synthase (GGPPS), and GGPP is further catalyzed to form *ent*-copalyl diphosphate (*ent*-CPP). *Ent*-CPP is the only precursor in the biosynthesis of all types of DAs,^17,18^ and it is further cyclized to form *ent*-kaurane and *ent*-atisane, which are aminated to obtain the corresponding DAs. The pathways for the formation of DAs from the amination of other diterpenoids lack the support of intermediates found in nature, but there are more reports of *ent*-atisane-type diterpenoids.^19^ The coexistence of *ent*-atisane-type diterpenoids and atisine-type DAs in plants from *Aconitum*, *Delphinium*, and *Spiraea*, such as atisenol isolated from *A. heterophyllum*^15,20^ and spiraminol from *Spiraea*,^14^ further supports the abovementioned biosynthetic pathway.

Atisine-type DAs play an important role in the biosynthesis of DAs and are some of the most important precursors of all of the other types of DAs. As shown in Fig. 1, the linkage of C-7 and C-20 on the atisine-type DA forms denudatine-type C_20_-DAs. The formation of the C-14–C-20 bond on atisine-type DA generates hetidine-type DAs, which can further generate hetisine-type DAs by linkage of the C-6–N bond.^21^ In addition, the fission of the C-19–N bond of hetisine-type DAs produces vakognavine-type DAs. Atisine-type DAs transform into veatchine-type DAs *via* 16(12 → 13)-*abeo*-shift rearrangement, which can produce napelline-type DAs *via* the formation of C-6–C-20 bond. In addition, C_19_-DAs are also biosynthesized from atisine-type DAs.^22^

## 3. Structure and classification

Among C_20_-DAs, atisine-type DAs have the simplest skeleton and possess a basic skeleton consisting of five six-membered rings, including four cyclohexane rings, A (C-1–C-5, C-10), B (C-5–C-10), C (C-8, C-9, C-11, C-12, C-15, C-16), D (C-8, C-9, C −11, C-12–C-14), and azacycle E (C-4, C-5, C-20, C-19, *N*). In addition, it may have an oxygen heterocycle F (C-5–C-7, *O*, C-20, C-10) or an oxazolidine ring G (*N*, C-19, O, C-21, C-22 or *N*, C-20, O, C-21, C-22) (Fig. 1). The fusion of rings is identical for all atisine-type DAs, *i.e.*, the A/B and E/F rings are *cis*-fused, and the A/E and B/C rings are *trans*-fused. In addition, according to single-crystal X-ray diffraction analysis of the corresponding atisine-type DAs, such as atisinium chloride, dihydroatisine, isoatisine, atisine 15-acetoxyazomethine, and 5-hydroxyazomethine,^23,24^ the six-membered cyclohexane rings A and B are in chair conformations, rings C and D are in boat conformations, and the E ring is usually in a chair conformation, but it can be distorted to a semichair conformation when an imine is formed.^25^

Atisine-type DAs also share some common structural characteristics. For example, most atisines possess a C-16 and C-17 exocyclic double bond, while the exocyclic double bond in a few atisines might be oxidized to form exocyclic methylene groups or be reduced to the angle methyl group. Compared with other types of DAs, this type of compound possesses fewer types and quantities of oxygen-containing substituents, mainly OH, OAc, and =O, with only 1 case of OBz (O-benzoyl) substitution and only 2 cases of 2-methylbutyryl (MeBu) substitution. Hydroxyl (OH) and OAc groups are the most common substituents on atisines, and they are usually located at C-15 or/and C-7. In addition, atisine-type DAs feature abundant substituents at the *N* atom, which distinguishes them from other types of DAs whose *N* atom is commonly substituted by an ethyl group. According to the form of the *N* atom, natural atisines can be divided into the amine subtype (A-1), the *N*,*O*-mixed acetal/ketal subtype (A-2), the amide/lactam subtype (A-3), the imine subtype (A-4), the quaternary ammonium salt (A-5), and the enamine subtype (A-6). In recent years, aconicatisulfonine-type rearranged atisines (A-7) and cyano-containing atisines (A-8) have also been discovered. In addition, some types of bis-DAs containing atisine units were found. Table S1† lists the names, subtypes, plant sources, and references of a total of 87 naturally occurring atisine-type DAs and 11 bis-DAs containing atisine units reported in recent decades. Herein, the structural features of atisine-type DAs are discussed by category.

### 3.1. The amine subtype

**Fig. 2:**
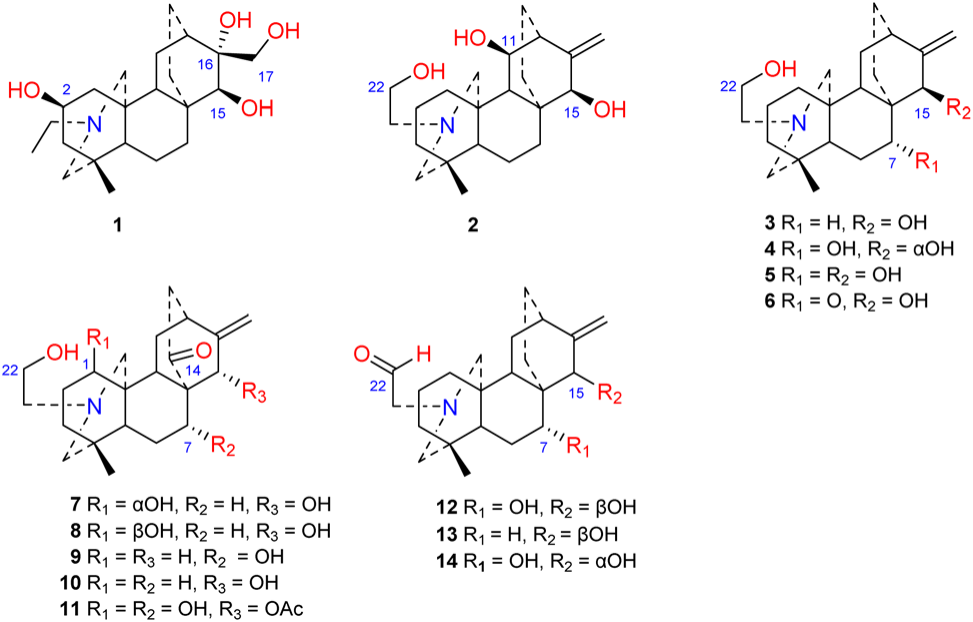
The amine subtype of atisines.

Approximately 14 amine-subtype atisines have been reported (Fig. 2). This type of compound is often substituted by a β-aminoethanol (*N*CH_2_CH_2_OH) group.^26,27^ The β-aminoethanol group may be oxidized to form an *N*CH_2_CHO group (*e.g.*, 12–14).^28–30^ In the amine subtype of atisines, the OH group is commonly substituted at C-7 and C-15. Cochleareine (1) isolated from *A. cochleare* possesses a vicinal triol system at C-15, C-16, and C-17.^31^

### 3.2. The *N*,*O*-mixed acetal subtype

**Fig. 3:**
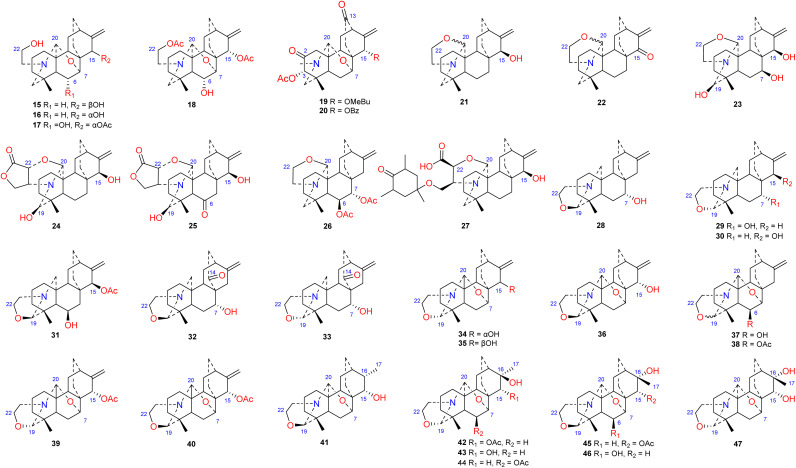
The *N*,*O*-mixed acetal subtype of atisines.

**Fig. 4:**
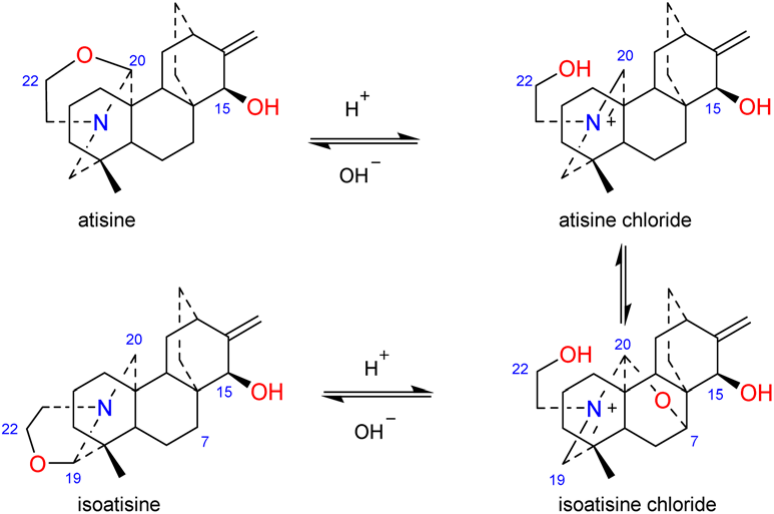
The transformation of atisine and isoatisine.

The *N*,*O*-mixed acetal compounds are the most numerous atisine-type DAs, with 33 compounds identified. These compounds may be further divided into three classes according to the type of *N*,*O*-mixed acetal group (Fig. 3). The first class of compounds includes six compounds (15–20) that feature an *N*–C-20–*O*–C-7 group.^32,33^ Among these compounds, compound 19 possesses a rare OMeBu substituent and compound 20 is the only atisine-type DA possessing an OBz substituent.^34^ The second class of 11 compounds (21–33) feature an oxazolidine ring, which may be formed by *N*–C-21–C-22–*O*–C-20 or *N*–C-21–C-22–*O*–C-19. Notably, these two types of oxazolidine rings are convertible under certain conditions. For example, atisine (21) may be slowly converted to isoatisine (30) in methanol at room temperature, and isoatisine (30) may be converted to atisine (21) after heating (Fig. 4). Compounds 24 and 25 from *D. chrysotrichum* possess an additional five-membered lactone ring connected to the oxazolidine ring,^35,36^ and compound 27 features a unique 1′,3′,5′-trimethyl-4′-oxocyclohexyloxy unit.^37^ The third class of compounds (34–47) has an oxazolidine ring and an *N*–C20–*O*–C7 functional group. Seven compounds (41–47) of this class possess a methyl group at C-17 with variable orientations.^38–40^

### 3.3. The amide/lactam subtype

**Fig. 5:**
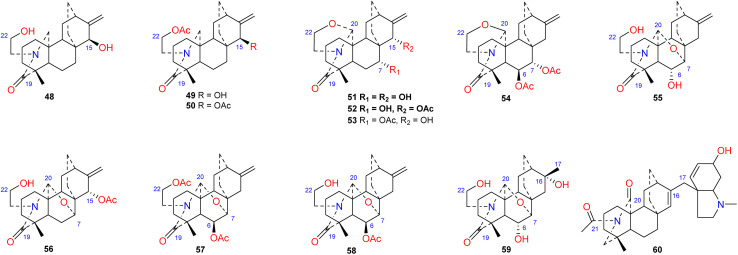
The amide/lactam subtype of atisines.

To the best of our knowledge, approximately 12 amide/lactam subtype of atisines have been reported (48–60) (Fig. 5). This type of compound generally has an *N*–C-19=*O* lactam group.^41–43^ Coryphidine (60) isolated from *A. coreanum* has carbonyl groups at C-20 and C-21, which generates an imide.^44^ Notably, a rare hexahydro-*N*-methyl indole fragment was linked to C-17 in its structure. The oxygen-containing substituent at C-6 of atisine-type DAs is generally β-oriented. However, spiramilactams A and B (55 and 59) from *S. japonica* var. *ovalifolia* possess an OH-6α substituent.^43^

### 3.4. The imine subtype

**Fig. 6:**
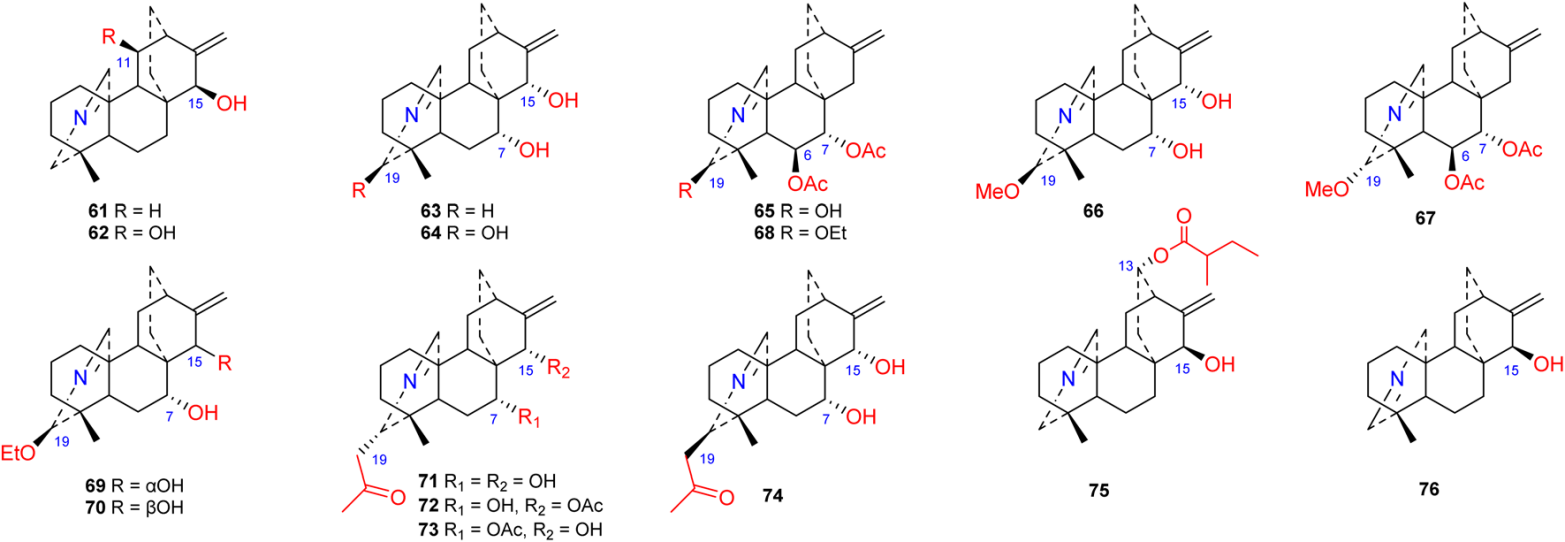
The imine subtype of atisines.

Approximately 16 compounds belonging to this subtype of atisines have been reported (61–76) (Fig. 6). Fifteen of these compounds possess an *N*=C-20 imine structure,^45–47^ and only one compound, 76, has an *N*=C(19) imine structure.^41^ C-19 in this subtype of compound is commonly oxygenated by OH, OMe or OEt groups. Compounds 71–74, which were isolated from *S. japonica* var. *acuminata*, possess an acetonyl substituent at C-19 and may be artifacts.^48^ The 13-(2-methylbutyryl)azitine (75) isolated from *D. scabriflorum* is oxygenated at the C-13 position and contains an OMeBu substituent.^41^

### 3.5. Quaternary ammonium salts and enamines

**Fig. 7:**
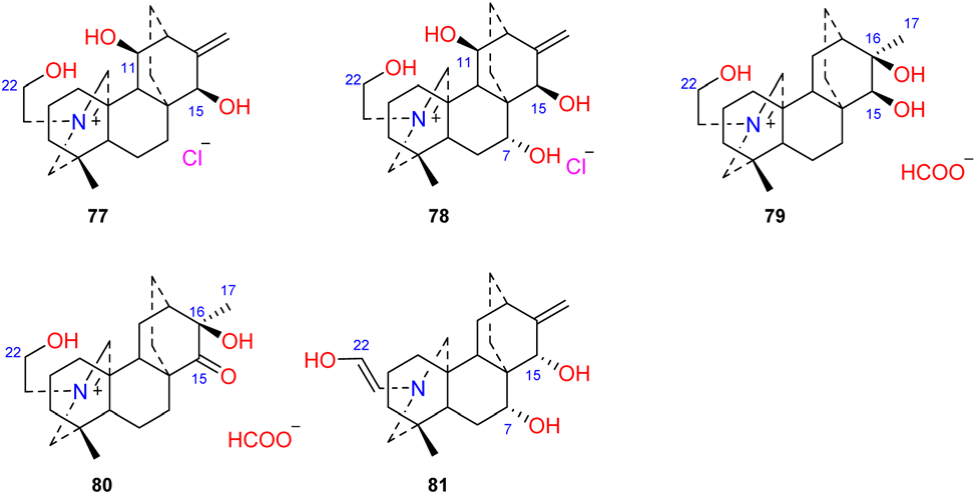
The quaternary ammonium salts and enamines of atisines.

Four atisines have been reported as quaternary ammonium salts (Fig. 7), including two hydrochloride salts, leucostomines A and B (77 and 78), which were isolated from *A. leucostomum*,^49^ and two formate salts, barpubesines A and B (79 and 80), which were obtained from *A. barbatum* var. *puberulum*.^26^ Uncinatine (81) isolated from *D. uncinatum* is a rare enamine with a Δ^21,22^ group.^50^

### 3.6. The rearranged subtype

**Fig. 8:**
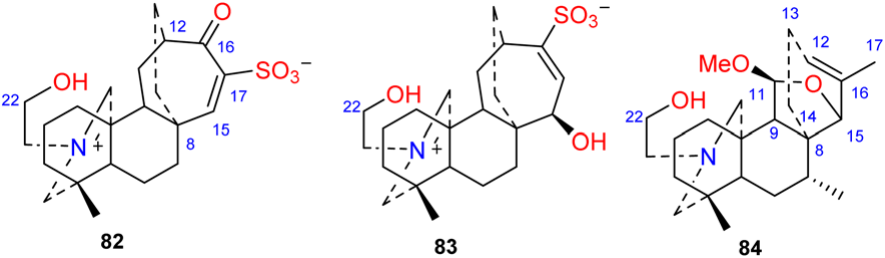
The rearranged subtype of atisines.

Two DAs, aconicatisulfonines A and B (82–83) (Fig. 8), with an unprecedented rearranged atisine skeleton, were reported from *A. carmichaelii*.^51^ The structure of compound 82 was verified using single-crystal X-ray diffraction. These two compounds are amphoteric compounds with sulfite and quaternary ammonium groups. Compounds 82 and 83 have a unique rearranged seven-membered C-ring, which may be the result of SO_3_H attacking the C-16 of atisine with a C-16–*O*–C-17 epoxy group, followed by Wagner–Meerwein rearrangement.^10^ Barpuberudine (84) has a rearranged novel atisine skeleton and was found in *A. barbatum* var. *puberulum*.^26^ Barpuberudine (84) features a C ring in which the C-11–C-12 bond is broken instead of a C-11–*O*–C-15 linkage, which forms a tetrahydrofuran ring. Barpuberudine (84) possesses a spiro D ring with a rearranged Δ^12,16^ group. Notably, anthriscifolsine A is a hetidine-type DA with a similar rearranged C/D ring, and it was isolated from *D. anthriscifolium* var. *majus*.^52^

### 3.7. The cyano-containing subtype

**Fig. 9:**
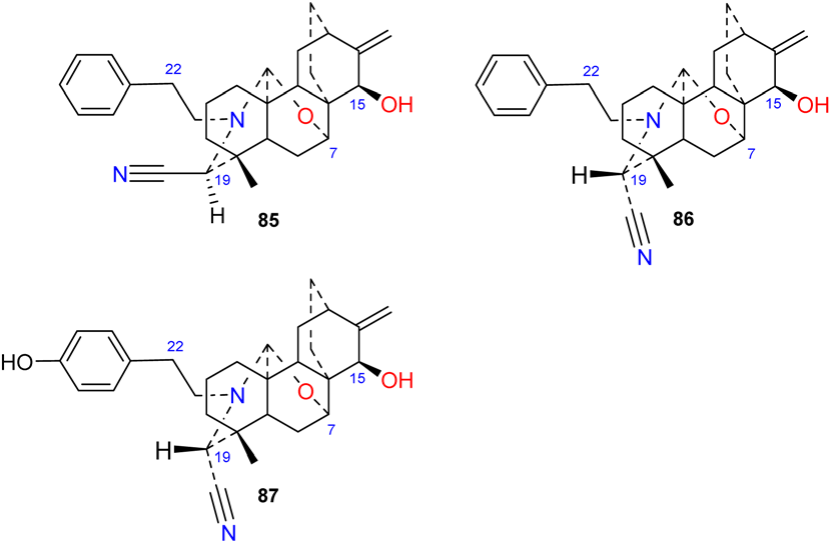
The cyano-containing subtype of atisines.

Brunonianines A–C (85–87) (Fig. 9) containing an unprecedented cyano substituent at C-19 were isolated from *D. brunonianum*.^53^ Brunonianines A and B (85 and 86) are a pair of epimers, and the structure of brunonianine A (85) was confirmed using single-crystal X-ray diffraction. Brunonianines A and B (85 and 86) have a phenethyl substituent on the *N* atom, and brunonianine C (87) has a *p*-hydroxyphenethyl group on the *N* atom. The phenethyl substituent of this type of compound may be formed by the amination of phenylalanine when atisane-type diterpenoids are transformed to atisine-type DAs, and the cyano group may be formed by glycine *via* the Mannich reaction.

### 3.8. Bis-DAs containing an atisine unit

**Fig. 10:**
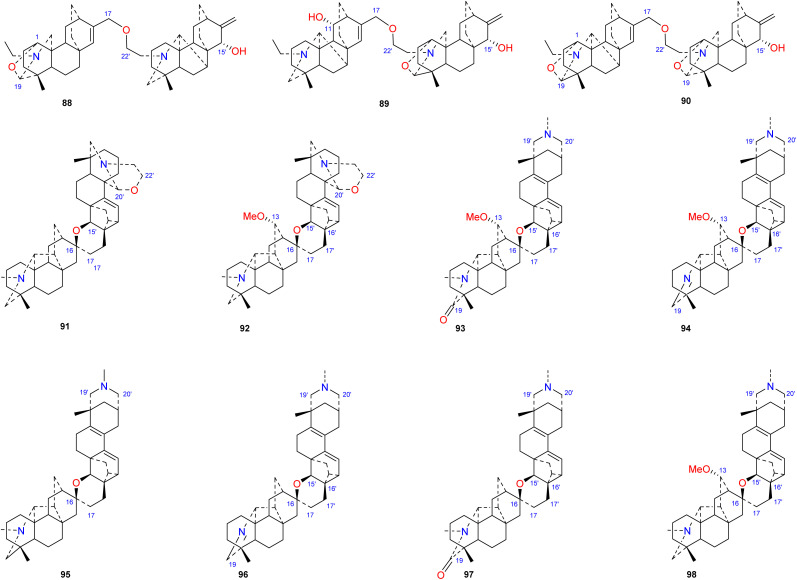
The Bis-DAs containing an atisine unit.

Bis-DAs are DA dimers formed by the condensation of two C_20_-DAs or one C_19_-DA and one C_20_-DA (Fig. 10). Twenty-three bis-DAs have been found in nature, 11 of which contain an atisine unit in their structures. These compounds can be further divided into 4 subtypes according to the construction units, namely, the atisine–denudatine subtype, the denudatine–atisine subtype, the hetidine–atisine subtype and the hetidine-rearranged atisine subtype.

Atisine–denudatine-type bis-DA is formed by linking atisine-type DA and denudatine-type DA *via* a C-17–*O*–C-22′ ether bond.

Bulleyanine B (88) is an atisine–denudatine bis-DA isolated from *A. bulleyanum* and is the only member of its subtype reported thus far.^54^ The denudatine–atisine type is created by the formation of an ether bond between C-17 in the denudatine-type DA and C-22′ in the atisine-type DA. Two compounds of this type have been reported, piepunine (89) isolated from *A. piperunense*^55^ and bulleyanine A (90) isolated from *A. bulleyanum*.^54^ Hetidine–atisine-subtype bis-DA is formed by linking C-16 and C-17 in a hetidine-type DA with C-15′, C-16′ and C-17′ in an atisine-type DA to form a tetrahydropyran ring. Only two compounds of this subtype of bis-DAs have been reported, staphisagrine (91) and staphisagnine (92), from *D. staphisagria*.^56^ The connection pattern of hetidine-rearranged atisine-type bis-DAs is analogous to that of hetidine–atisine-type bis-DAs. The difference is that the A ring in the atisine unit is rearranged, the C-20–C-10 bond is rearranged to C-20–C-2. There are six components (93–98) in this category, all of which were isolated from *D. staphisagria*.^57,58^

## 4. Distribution

Atisine-type DAs are distributed in plants of two families and five genera, namely, *Aconitum*, *Delphinium*, *Consolida*, and *Thalictrum* in the Ranunculaceae family and *Spiraea* in the Rosaceae family (Table 1). Only eight atisines have been found in *Consolida*,^28,59–61^ and only one atisine-type DA has been isolated from *Thalictrum*.^62^*Spiraea* are the richest source of atisines, with a considerable number coming from *S. japonica*. Thirty-three atisines have been isolated from the genus *Delphinium*. Among the 11 bis-DAs that have been isolated, 8 were isolated from *D. staphisagria*.

**Table 1:** The distribution of atisine-type DAs

Family	Genus	Number of atisine-type DAs
Ranunculaceae	*Aconitum*	28
	*Delphinium*	33
	*Consolida*	8
	*Thalictrum*	1
Rosaceae	*Spiraea*	47

In terms of the distribution of individual compounds, most atisine-type DAs were reported from only one certain species. This means that these compounds may be used as chemical markers for this plant to assist in its taxonomic identification. The most widely distributed compound is atisine (21), which has been isolated from 26 species. Ajaconine (15) was found in 16 species. However, they were all isolated from *Aconitum* and *Delphinium*. These two compounds may be precursors of other atisine-type DAs and other types of DAs, such as C_19_-lycoctonine-type DAs, in *Aconitum* and *Delphinium* plants.^63^

## 5. Biological activity

### 5.1. Antitumor activity

Some atisine-type DAs significantly inhibited tumor cell proliferation *in vitro*. Honatisine (27) isolated from *D. honanense* inhibited the proliferation of the human breast cancer cell line MCF-7, with an IC_50_ value of 3.16 μM, which was better than that of the positive control etoposide (IC_50_, 7.53 μM).^37^ Delphatisine C (25) isolated from *D. chrysotrichum* showed significant *in vitro* tumor cytotoxicity against the human lung adenocarcinoma cell line A549, with an IC_50_ of 2.36 μM, which was comparable to that of the positive control etoposide. ^36^ Li *et al.* reported that the cyano-containing compounds brunonianines A–C (85–87) inhibited the proliferation of Caco-2, H460 and Skov-3 tumor cells (Table 2). Brunonianines B and C (86 and 87) inhibited the proliferation of Caco-2 cells to a level comparable to that of the positive control hydroxycamptothecin, while 86 had a stronger inhibitory effect on the proliferation of Skov-3 cells than the positive control. The difference in the antitumor activities of brunonianines A and B can be attributed to the stereochemistry of the cyano-containing C-19, with the *S* configuration being significantly more potent than the *R* configuration. Further studies revealed that brunonianine B (86) arrested the Skov-3 cell cycle in the G2/M phase, reduced the mitochondrial membrane potential, and induced the apoptosis of Skov-3 cells to inhibit cell proliferation. This antitumor effect may be partially due to its inhibition of cell movement to prevent tumor metastasis. Western blot analysis revealed that it induced cell apoptosis *via* the Bax/Bcl-2/caspase-3 signalling pathway.^53^

**Table 2:** The cytotoxicity of natural atisines against human tumor cells (IC_50_, μM)

Compounds	A549	Caco-2	H460	Skov-3
Honatisine (27)	3.16	—	—	—
Delphatisine C (25)	2.36	—	—	—
Brunonianine A (85)	>50	15.5	25.4	>50
Brunonianine B (86)	>50	3.14	19.6	2.20
Brunonianine C (87)	>50	2.41	28.3	6.88

**Fig. 11:**
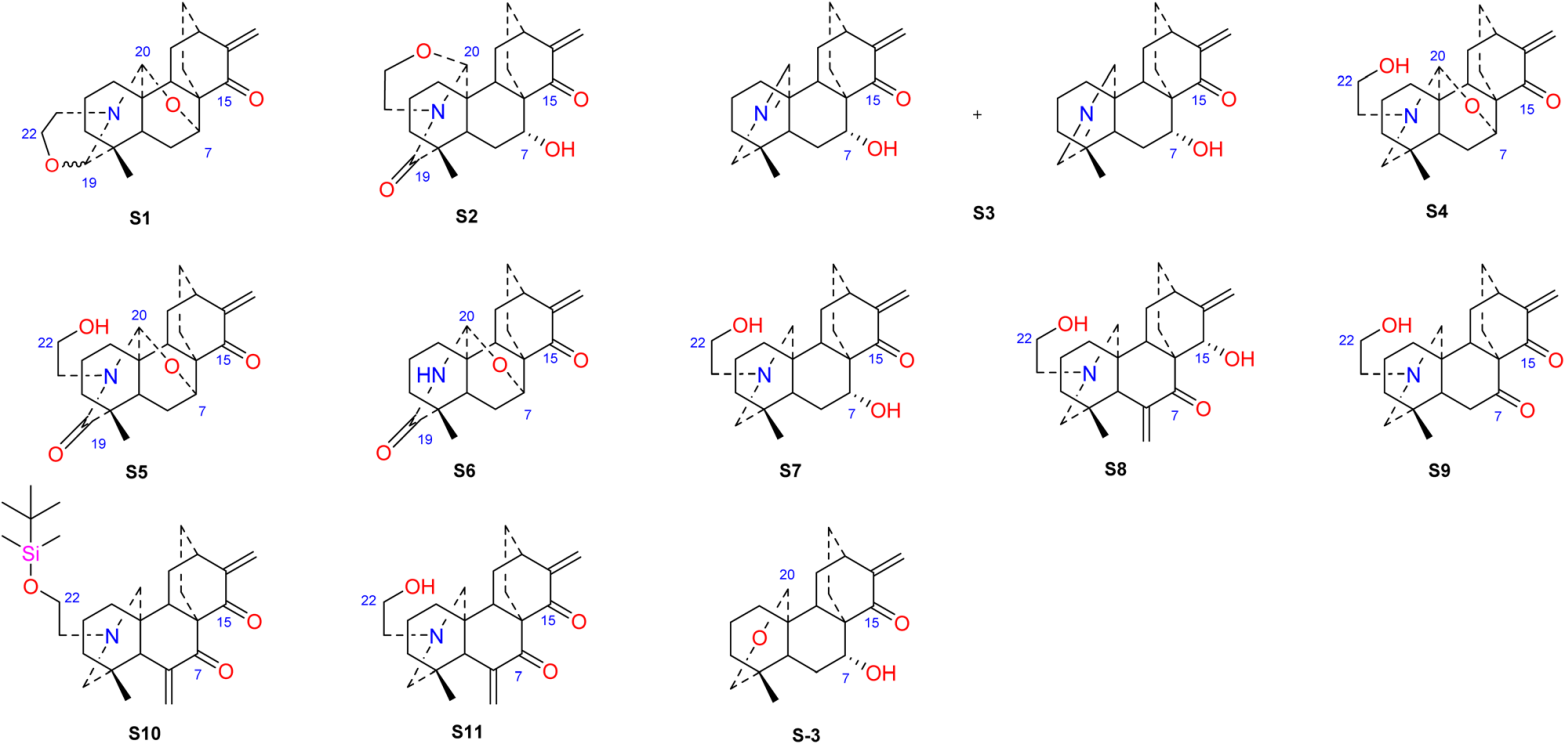
The structures of derivatives of spiramines C and D (S1–S11).

**Fig. 12:**
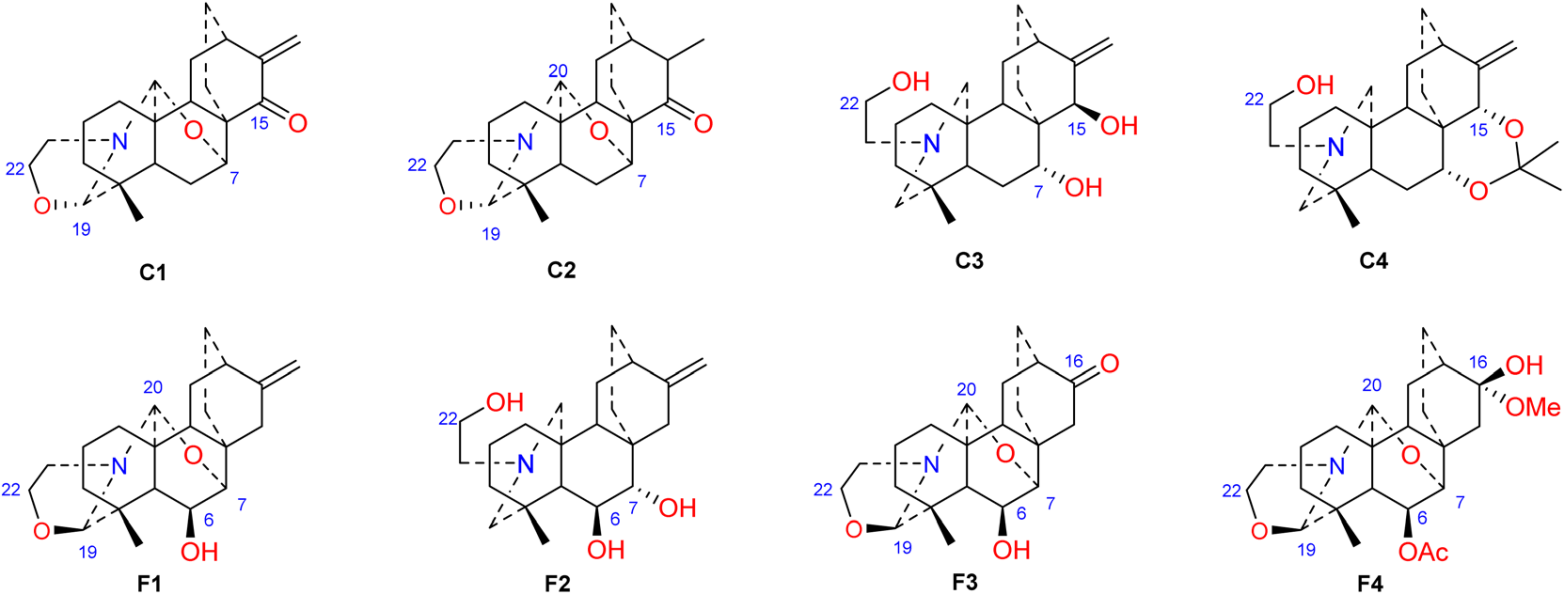
The structures of derivatives of spiramines C and F (C1–C4, F1–F4).

Hao *et al.* performed *in vitro* antitumor activity studies on 11 structurally modified derivatives (S1–S11) of spiramines C and D (34 and 35) (Fig. 11).^64^ These compounds inhibited the proliferation of a series of tumor cells, including HL-60, SMMC-7721, A-549, MCF-7, and SW-480 cells (Table 3). The toxicity of the S1 and S2 derivatives against the five tumor cell lines was greater than that of the S-3 and cisplatin positive controls, which showed the potential for their development as anticancer drugs. All the derivatives induced the apoptosis of Bax/Bak double knockout murine embryonic fibroblasts (Bax^−/−^/Bak^−/−^ MEFs) to different degrees, while S1, S2, S9 and S11 exhibited the strongest activities. Their IC_50_ values were 2.243 μM, 3.377 μM, 4.524 μM and 1.814 μM, respectively, and the IC_50_ of the positive control S-3 was 1.736 μM. The induction of Bax- and Bak-deficient cells and cancer cell apoptosis by S1, S2, S9 and S11 was also verified by annexin V/PI and caspase activation experiments. Compared to the positive control adriamycin, compounds S2, S3 and S6 (Table 4) had significant inhibitory effects on the proliferation of wild-type MCF-7 cells and adriamycin (ADR)-induced multidrug-resistant MCF-7 cells (MCF-7/ADR), which indicated that spiramine derivatives may overcome drug resistance. Preliminary structure–activity relationship studies showed that the oxazolidine ring was essential for cytotoxicity (Fig. 12). However, the C-7–*O*–C-20 ether bond is unfavorable for cytotoxicity. Atisine derivatives bearing double “Michael response receptor” groups outperformed derivatives with mono “Michael response receptor” group in inducing the apoptosis of Bax^−/−^/Bak^−/−^ cells and cytotoxicity in tumor cell lines.

**Table 3:** The IC_50_ values of cytotoxicity of spiramine derivatives (μM)

Compounds	HL-60	SMMC-7721	A-549	MCF-7	SW-480
S1	1.34	1.04	2.03	0.67	1.97
S2	2.73	2.63	3.09	1.12	2.81
S3	4.03	5.02	8.45	3.12	5.47
S4	19.27	23.63	18.57	10.35	13.99
S5	18.59	18.35	17.94	13.23	12.41
S6	18.55	15.73	19.02	15.73	14.19
S7	7.95	11.16	9.92	16.57	14.41
S8	4.82	1.48	3.04	6.98	9.84
S9	14.32	15.19	9.24	15.70	11.82
S10	1.39	2.63	1.55	2.90	2.78
S11	6.61	11.52	6.50	3.57	2.81
S-3	6.36	3.96	3.22	3.25	3.58
Cisplatin	3.08	10.20	9.08	17.48	11.99

**Table 4:** The IC_50_ values against MCF-7 and MCF-7/ADR for compounds S2, S3 and S6 (μM)

Compounds	MCF-7	MCF-7/ADR	Resistant factor
S2	5.287	8.802	1.66
S3	4.730	6.759	1.43
S6	20.167	28.319	1.40
Adriamycin	0.077	68.434	888.75

### 5.2. Anti-platelet aggregation effect

Thrombotic disease is a common cardiovascular disease that seriously harms human health.^65^ Platelet aggregation is a key factor in the development of thrombi, and the discovery of anti-platelet aggregation components from natural products is highly important.^66^ Hao *et al.* reported that the atisine-type DA spiramine Q (43) had selective *anti*-arachidonic acid-induced platelet aggregation activity, and its inhibitory effect was stronger than that of aspirin.^67^ Hao *et al.* subsequently evaluated the antiplatelet aggregation effects of six atisine-type DAs and their eight structurally modified derivatives on the basis of arachidonic acid, adenosine diphosphate (ADP) and platelet-activating factor (PAF).^67,68^ Among the atisine-type DAs tested, 12 compounds (Table 5) significantly inhibited PAF-induced platelet aggregation in a concentration-dependent manner but had no effect on the aggregation induced by ADP or arachidonic acid, which showed selective inhibition. The antiplatelet aggregation effects of hetisine-type DAs, which are structurally similar to atisine-type DAs, are poorly selective.^69^ Among the screened compounds, only spiramine C1 (Fig. 12) concentration-dependently inhibited platelet aggregation induced by PAF, ADP, and arachidonic acid, which indicated a nonselective antiplatelet aggregation effect. The inhibitory effect of spiramine C1 on arachidonic acid was comparable to that of aspirin. Preliminary SAR analysis (Fig. 11) showed that an oxazolidine ring was critical for their antiplatelet aggregation effect. The antiplatelet aggregation effect of atisines was strongly affected by substitution at C-15. Atisine-type DAs are a novel class of selective antiplatelet aggregation agents. Although they are less potent than the known PAF receptor-specific antagonist ginkgolide B, more effective derivatives may be obtained *via* structural modification.

**Table 5:** The anti-platelet aggregation effect of atisine-type DAs (IC_50_, μM)

Compd.	PAF	Arachidonic acid	ADP
Spiramine Q (43)	—	18.7	—
Spiramine A (39)	6.7	—	—
Spiramine C (34)	32.6	—	—
Spiradine F (38)	138.9	—	—
Spiramine Z-2 (28)	58.4	—	—
Deacetylspiramine F (16)	309.3	—	—
Spiramine V (53)	87.1	—	—
Spiramine C1	30.5	29.9	56.8
Spiramine C2	53.3	—	—
Spiramine C3	147.5	—	—
Spiramine C4	172.6	—	—
Spiradine F1	82.0	>240	—
Spiradine F2	>240	—	—
Spiradine F3	92.9	—	—
Spiradine F4	110.7	—	—
Ginkgolide B	0.43	—	—
Aspirin	—	35.2	—

### 5.3. Biological control effects

Some plants of the genera *Aconitum* and *Delphinium* may be used as pesticides, which supports the potential biocontrol properties of these main ingredients. Plant virus diseases are a group of important agricultural diseases whose harm to cultivated crops is second only to fungal diseases. Tobacco mosaic virus (TMV) is a model virus for plant virus research, with a wide range of host plants and a worldwide distribution. It can infect a variety of important economic crops, such as tomato, potato, pepper, cucumber, and tobacco, and cause major economic loss.^70^ The discovery of active substances against TMV has important practical significance. Hao *et al.* used the half-leaf method to screen a series of atisine-type DAs isolated from *S. japonica* var. *acuminata* for anti-TMV effects, including protective effects and healing effects. The experimental results showed that most of the compounds exhibited greater activity than did the positive control ningnanmycin. The inhibition rates of some compounds, such as 38, 65, 67 and 68 (Table 6), were 73.5–92.9% at 100 μg mL^−1^. Preliminary SAR analysis showed that atisine-type DAs with an imine at C-20 and an acyl substituent at C-6 had better antiviral effects. Compounds 4 and 47 also exhibited healing effects that were superior to those of ningnanmycin.

**Table 6:** *In vivo* antiviral activities of atisine-type DAs against TMV (inhibition rate at 100 μg mL^−1^, %)

Compd.	Protective effect	Curative effect	Compd.	Protective effect	Curative effect
Spirimine A (63)	<20	<20	Spiramine C/D (34/35)	<20	53.94
Spirimine A (67)	92.91	41.3	Spiradine F (38)	85.34	48.07
Spiramine C2 (41)	41.74	<20	Spiramine P (47)	51.55	76.98
Spiratine B (65)	87.08	20.84	Spiramine F (17)	51.71	<20
Spiramine Z (68)	73.52	46.50	Triol (4)	<20	62.85
Spiramine A/B (39/40)	<20	43.81	Ningnanmycin	48.20	50.72

**Fig. 13:**
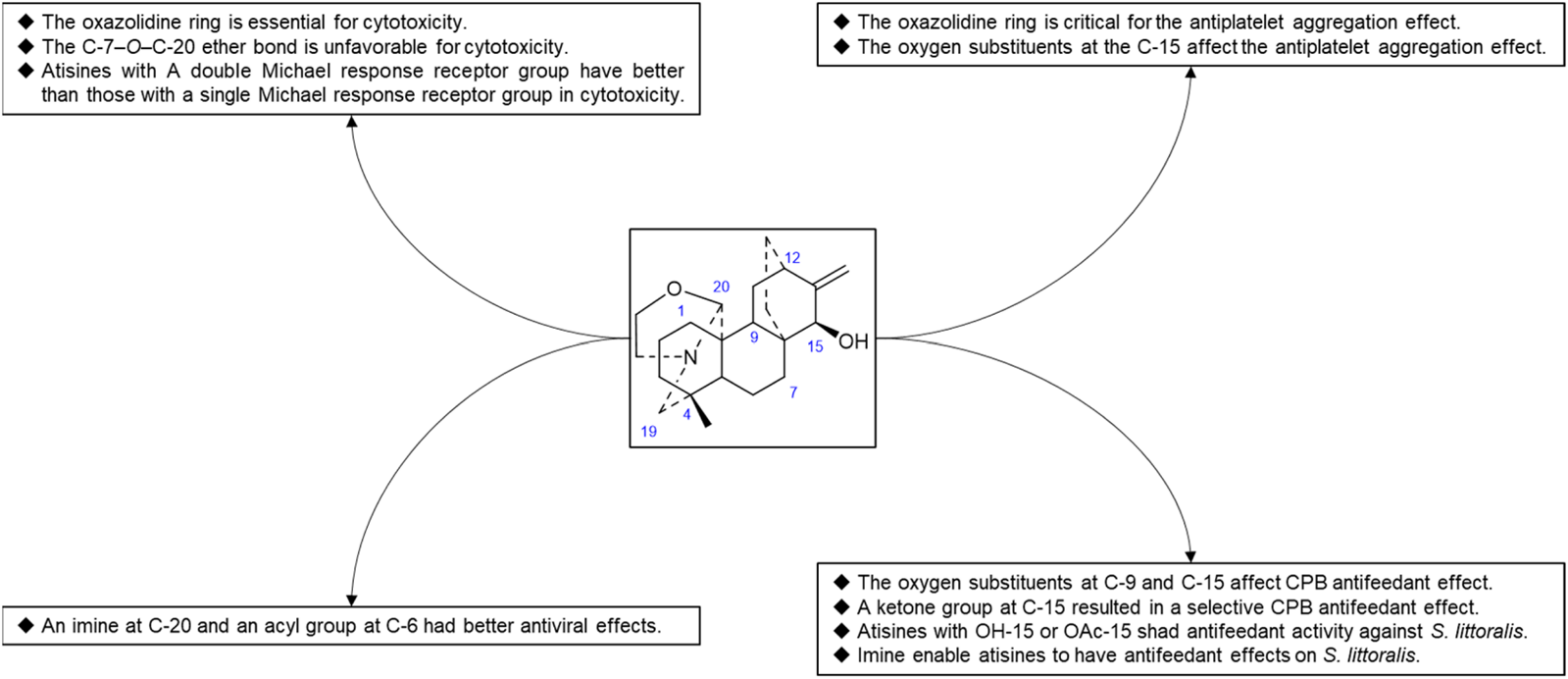
The SAR of atisine-type DAs.

Reina *et al.* studied the insecticidal effects of more than 70 DAs, including nine natural atisines and two synthetic derivatives called alkaloids A and B.^71,72^ As shown in Table 7, the antifeeding effect of the satisfactory-type compounds was structure- and species dependent. Except for compounds 48, 50 and 61, most of the atisines tested had antifeedant effects on the potato beetle *Leptinotarsa decemlineata* (CPB), with EC_50_ values ranging from 3.9 to 6.9 μg cm^−2^. Only six atisines showed antifeeding effects on *S. littoralis*, with EC_50_ values ranging from 0.1 to 8.2 μg cm^−2^. In general, the antifeeding effect of atisines was weaker than that of C_19_-DAs. Compound 48 exhibited the strongest antifeeding effect on *S. littoralis* (EC_50_ of 0.1 μg cm^−2^), but it was ineffective against CPB. Compound 3, which had a strong effect on CPB (EC_50_ of 2.9 μg cm^−2^), was also effective against *S. littoralis*. Reina *et al.* further tested the effects of DAs on insect Sf9 and CHO cells and *Trypanosoma cruzi* epimastigotes, which are two biological models lacking neurotransmission. Compounds 48 and 21 exhibited Sf9 cytotoxicity, and compound 50 exhibited toxicity against *T. cruzi* epimastigotes. These toxic effects suggest that the mechanism of action of atisines is different from that of C_19_-DAs. C_19_-DAs have strong neurotoxicity and specifically act on voltage-gated Na channels. SAR analysis revealed that the antifeedant effect of atisines on CPB was related to oxygen substitutions at C-9 and C-15, and the presence of a ketone group at C-15 resulted in a selective CPB antifeedant (*e.g.*, compounds 16 and 17). Atisines with OH-15 or OAc-15 substituents and without OH-7 substituents had antifeedant activity against *S. littoralis*. Imines also enable atisines to exhibit antifeedant effects on *S. littoralis* (Fig. 13).

**Table 7:** Antifeedant activities of atisine-type DAs

Compd.	*L. decemlineata* (EC_50_, μg cm^−2^)	*S. littoralis* (EC_50_, μg cm^−2^)	*T. cruzi* (EC_50_, μg mL^−1^)	Sf9 cells (EC_50_, μg mL^−1^)
9-Oxodihydroatisine (48)	>50	0.1	>100	11.4
15,22-*O*-Diacetyl-19-oxo-dihydroatisine (50)	>50	6.1	23.0	>100
Dihydroajaconine (5)	5.0	>50	>100	>100
15-*Epi*-dihydroatisine (3)	2.9	>50	>100	>100
Atisinium chloride (21)	3.4	2.4	>100	38.2
7α-Hydroxyisoatisine	3.4	>50	>100	>100
Ajaconine (15)	5.1	8.2	>100	>100
Azitine (61)	>50	1.1	>100	>100
Isoazitine (76)	6.9	4.1	>100	>100
Alkaloid A	5.4	≈50	>100	>100
Alkaloid B	3.6	>50	>100	>100

These DAs have also been screened for their antiparasitic effects (Table 8), and only three atisine-type Das, 50, 61 and 76, exhibited strong *in vitro* leishmanicidal activity against the promastigote *L. infantum*. Among these DAs, 76 had the strongest effect, followed by 61, and 50 had the poorest effect.^73^ Reina *et al.* used an *in vitro* culture system of mammalian host cells (Vero) infected with *Trypanosoma cruzi* to screen anti-*T. cruzi* agents from a total of 43 C_19_-DAs and 21 C_20_-DAs.^74^ Among the 64 DAs tested, only five C_20_-DAs were active against *T. cruzi* epimastigotes. Atisinium chloride showed a strong inhibitory effect similar to that of the reference drug benznidazole. These compounds also attenuated the ability of the parasite to invade mammalian cells, and its ability to replicate and transform into trypomastigotes in the cells and were nontoxic to host cells (Vero cells) (compound 21, IC_50_ > 300). These studies highlight the ideal insecticidal effect of atisines, and their significant effect and unique molecular selectivity are worthy of further investigation. The use of atisine-type DAs as lead compounds *via* structural modification will likely result in compounds with stronger activity and provide new medicinal resources for the prevention and treatment of protozoal infections.

**Table 8:** Antiparasitic effects of atisine-type DAs

	*L. infantum* promastigotes (IC_50_, μg mL^−1^)			*T. cruzi* epimastigotes (IC_50_, μg mL^−1^)		
	24 h	48 h	72 h	24 h	48 h	72 h
15,22-*O*-Diacetyl-19-oxo-dihydroatisine (50)	24.58	15.74	12.80	—	—	98.36
Azitine (61)	26.30	15.35	10.12	—	—	67.74
Isoazitine (76)	13.38	9.70	7.39	—	—	>100
Atisinium chloride (21)	—	—	—	13.91	9.37	5.46
Pentostam	—	—	11.32	—	—	—
Benznidazole	—	—	—	—	—	4.12

### 5.4. Anti-inflammatory effect

Plants of the genera *Aconitum*, *Delphinium*, and *Spiraea* are widely used to treat inflammation-related diseases, such as arthritis and pain, which suggests that their main components, DAs, have anti-inflammatory effects.^6,75^ Bis-DA bulleyanine A (90) isolated from *A. bulleyanum* had a significant anti-inflammatory effect *in vitro*, and the inhibition rate of NO (nitric oxide) induced by LPS (lipopolysaccharide) in RAW264.7 macrophages at 40 μM was 74.60%.^54^ Forrestline F (62), which is a novel compound isolated from *D. forrestii* var. *viride*,^42^ also showed significant anti-inflammatory effects *in vitro*, with an IC_50_ value of 9.57 μM for the inhibition of NO production in RAW264.7 macrophages induced by LPS. Forrestline F (62) also inhibited the expression of inflammatory cytokines, including TNF-α (tumor necrosis factor alpha), IL-1β (interleukin 1β), and IL-6, and downregulated the expression of iNOS (inducible nitric oxide synthase) and COX-2 (cyclooxygenase-2) in RAW264.7 macrophages induced by LPS. Forrestline F (62) exerted anti-inflammatory effects *via* the inhibition of the NF-κB/MAPK (nuclear factor kappa-B/mitogen-activated protein kinase) and Nrf2/HO-1 (nuclear factor erythroid 2-related factor 2/heme oxygenase-1) signalling pathways, and it inhibited MAPK (including p-p38, p-ERK and p-JNK) and NF-κB phosphorylation of p65 and upregulated HO-1 expression *via* Nrf2 nuclear translocation, which reduced ROS (reactive oxygen species) accumulation.^76^

### 5.5. Analgesic effect

The rearranged-type atisines aconicatisulfonines A and B (82 and 83) exhibited significant analgesic effects in acetic acid-induced writhing experiments in mice.^51^ At doses of 0.1, 0.3 and 1.0 mg kg^−1^ (i.p.), the writhing response of mice decreased by 23.3%, 43.2%, and 50.3%, respectively, for compound 82 and by 46.6%, 64.7% and 75.7%, respectively, for compound 83, compared to a 66.8% writhing reduction at 0.3 mg kg^−1^ (i.p.) for the positive control morphine.^51^

### 5.6. Antiarrhythmic effect

Dzhakhangirov *et al.* screened the antiarrhythmic effects of more than 100 DAs using a model of aconitine-induced arrhythmia in anesthetized rats, including four atisines, atidine (6), dihydroatisine (3), atisine (21), and isoatisine (30).^77,78^ Atidine (6) and dihydroatisine (3) exhibited certain antiarrhythmic effects with ED_50_ values of 5 mg kg^−1^ and 1 mg kg^−1^, respectively. However, when combined with representative compounds of other types of DAs, such as the C_18_-DAs lappaconitine (ED_50_, 0.05 mg kg^−1^) and deacetyllapaconitine (ED_50_, 0.05 mg kg^−1^) and the C_19_-DA 6-benzoylheteratisine (ED_50_, 0.035 mg kg^−1^), the antiarrhythmic effects of atisine-type DAs were not sufficient.^79^ Therefore, further screening of this type of alkaloid was not performed.

### 5.7. Cholinesterase inhibition

Ajaconine (15) isolated from *D. chitralense* inhibited cholinesterase with IC_50_ values for AChE (acetylcholinesterase) and BchE (butyrylcholinesterase) of 12.61 μM and 10.18 μM, respectively.^80^ A mechanism-based kinetic study showed that it was a competitive inhibitor of AChE and BchE. Heterophyllinine-B (31) isolated from *A. heterophyllum* from Turkey had selective cholinesterase inhibition with an IC_50_ value of 40.63 μM for BchE inhibition but no inhibitory effect on AChE.^81^

### 5.8. Toxicity

To date, only a few atisine-type DAs have been investigated for their acute toxicity in mice. The LD_50_ (i.v.) values for compounds 3, 6, 21, 30, and 60 are 38 mg kg^−1^, 58 mg kg^−1^, 9 mg kg^−1^, 8 mg kg^−1^, and 20 mg kg^−1^, respectively.^77^ In general, the toxicity of atisine-type DAs was lower than that of aconitine-type Das such as aconitine (LD_50_, 0.125 mg kg^−1^) and 3-acetylaconitine (LD_50_, 0.27 mg kg^−1^),^82^ which highlights their potential in drug discovery.

## 6. Conclusion

A total of 87 atisine-type DAs and 11 bis-DAs containing atisine units have been reported from five genera in two families. The genus *Spiraea* in the Rosaceae family may be the richest resource for atisine-type DAs, followed by the genera *Delphinium* and *Aconitum* in the Ranunculaceae family. Among the reported atisine-type DAs, several possess unprecedented skeletons. Atisine-type DAs have a wide range of biological activities, including antitumor, antiplatelet aggregation, biological control, anti-inflammatory, analgesic, antiarrhythmic, and cholinesterase inhibitory effects. The antiparasitic effect of atisine-type DAs is more prominent than the other types of DAs, which highlights their potential in antiparasitic drug discovery. In summary, the high chemical and biological diversity of atisine-type DAs indicate their great potential as a vast resource for drug discovery.

## Data availability

All relevant data are within the manuscript and its additional files.

## Author contributions

Jiaqi Zheng: writing – original draft, writing – review and editing, supervision, visualization. Hongjun Jiang: writing – review and editing. Yuanfeng Yan: supervision, writing – review and editing. Tianpeng Yin: resources, supervision, writing – review and editing, funding acquisition.

## Conflicts of interest

The authors declare no conflict of interest.

## Supplementary Material

RA-014-D4RA03305A-s001
