# Supplementary material for: Overview of the chemistry and biological activities of natural atisine-type diterpenoid alkaloids
Source: RSC Adv. 2024 Jul 22;14(32):22882–93. doi: 10.1039/d4ra03305a (PMC11261430; doi:10.1039/d4ra03305a)
Supplement: RA-014-D4RA03305A-s001 [file RA-014-D4RA03305A-s001.pdf]

Electronic Supplementary Material (ESI) for RSC Advances.
This journal is © The Royal Society of Chemistry 2024

Electronic Supplementary Information

Overview of the chemistry and biological activities of natural atisine-type diterpenoid alkaloids

Jiaqi Zheng, Hongjun Jiang, Yuanfeng Yan, Tianpeng Yin*

Table 1S. Naturally occurring atisine-type diterpenoid alkaloids

No	Name	Plant source	Ref.
atisine-type C_{20}-diterpenoid alkaloids			
**1**	cochleareine	*A. cochleare*	[1]
**2**	barpubesine C	*A. barbatum* var. *puberulum*	[2]
**3**	dihydroatisine	*A. zeravschanicum*	[3]
		A. heterophyllum	[4]
		A. coreanum	[5]
		A. tanguticum	[6]
		C. hellespontica	[7]
		D. staphisagria	[8, 9]
**4**	triol	*S. japonica* var. *acuminate*	[10, 11]
**5**	dihydroajaconine	*A. gymnandrum*	[12]
		A. heterophyllum	[13]
		C. ambigua	[14]
		C. orientalis	[15]
		D. ajacis	[16]
		S. japonica var. *acuminata*	[17]
**6**	atidine	*A. anthoroideum*	[18]
		A. heterophyllum	[19]
		A. rotundifolium	[20]
		A. zeravschanicum	[21]
**7**	beiwusine A	*A. kusnezoflii*	[22]
**8**	beiwusine B	*A. kusnezoflii*	[22]
**9**	spiramine G	*S. japonica* var. *acuminata*	[23, 24]
**10**	spiramine H	*A. kusnezoffii*	[22]
		S. japonica var. *stellaris*	[25]
		S. japonica var. *acuminata*	[23]
**11**	spiramine I	*S. japonica* var. *acuminata*	[23]
**12**	consorientaline	*C. orientalis*	[26]
**13**	chellespontine	*A. naviculare*	[27]
		A. rotundifolium	[20]
		C. hellespontica	[7]
		C. raveyi	[28]
		D. staphisagria	[29]
		D. peregrinum	[30]
**14**	spiratine A	*S. japonica* var. *acuta*	[31]
**15**	ajaconine	*A. anthoroideum*	[18]
		A. rotundifolium	[20]
		A. stenocarpa	[32]
		C. raveyi	[28]
		C. aconiti	[33]

		C. ambigua	[14]
		C. axilliflora	[34]
		C. hohenackeri	[35]
		C. oliveriana	[36]
		C. orientalis	[37]
		D. ajacis	[38]
		D. carolinianum	[39]
		D. chitralense	[40]
		D. brunonianum	[41]
		D. delavayi	[42]
		D. elatum	[43]
		D. tatsienense	[44]
		D. virescens	[45]
**16**	deacetylspiramine F	*D. ajacis*	[46]
		S. japonica var. *ovalifolia*	[47]
**17**	spiramine F	*S. japonica* var. *acuminata*	[23, 48]
		S. japonica var. *acuta*	[49]
		S. japonica var. *ovalifolia*	[50]
		S. japonica var. *stellaris*	[25]
**18**	spiramine E	*S. japonica* var. *acuminata*	[24]
**19**	ouvrardiandine A	*A. ouvrardianum*	[51]
**20**	ouvrardiandine B	*A. ouvrardianum*	[51]
**21**	atisine	*A. anthora*	[52]
		A. anthoroideum	[53]
		A. tanguticum	[54, 55]
		A. bulleyanum	[56]
		A. coreanum	[5, 57]
		A. gigas	[58]
		A. gymnandrum	[12, 59]
		A. heterophylloides	[60]
		A. heterophyllum	[61, 62]
		A. napellus	[63]
		A. naviculare	[64]
		A. orochryseum	[65]
		A. palmatum	[66]
		A. racemulosum	[67]
		A. rotundifolium	[20]
		A. septentrionale	[68]
		A. taipaicum	[69]
		A. tanguticum	[70]
		A. vaginatum	[71]
		A. zeravschanicum	[21]
		C. regalis	[72]
		D. albocoeruleum	[73]
		D. honanense	[74]
		D. peregrinum	[30] [75]
		D. virgatum	[76]

		D. staphisagria	[8]
**22**	atisinone	*A. heterophyllum*	[77]
**23**	delphatisine A	*D. chrysotrichum*	[78]
**24**	delphatisine B	*D. chrysotrichum*	[78]
**25**	delphatisine C	*D. chrysotrichum*	[79]
**26**	Spiramine Z-1	*S. japonica* var. *ovalifolia*	[80]
**27**	honatisine	*D. honanense*	[74]
**28**	spiramine Z-2	*S. japonica* var. *ovalifolia*	[80]
**29**	spiramine Z-3	*S. japonica* var. *ovalifolia*	[80]
**30**	isoatisine	*A. anthora*	[52]
		A. coreanum	[81, 82]
		A. naviculare	[64]
		A. heterophyllum	[77]
		A. koreanum	[83]
		A. palmatum	[84]
		A. richardsonianum	[85]
		A. rotundifolium	[86]
		A. zeravschanicum	[21, 87]
		C. raveyi	[28]
		D. honanense	[88]
		D. honanense	[74]
**31**	heterophyllinine B	*A. heterohyllum*	[4]
**32**	spiramidine A	*S. japonica* var. *ovalifolia*	[89]
**33**	spiramidine B	*S. japonica* var. *ovalifolia*	[89]
**34**	spiramine C	*S. japonica* var. *acuminata*	[11] [17]
		S. japonica var. *stellaris*	[25]
		S. japonica var. *acuta*	[90]
		S. japonica var. *ovalifolia*	[50]
**35**	delphatisine D	*D. chrysotrichum*	[91]
**36**	spiramine D	*S. japonica var. ovalifolia*	[47]
		S. japonica var. *acuminata*	[11, 17]
		S. japonica var. *stellaris*	[25]
		S. japonica var. *acuta*	[90]
**37**	spiradine E (spiradine G)	*S. japonica*	[92]
**38**	spiradine F	*S. japonica* var. *acuminate*	[10]
		S. japonica var. *acuta*	[93]
		S. japonica var. *glabra*	[94]
**39**	spiramine A	*S. japonica* var. *acuminata*	[11]
		S. japonica var. *stellaris*	[25]
		S. japonica var. *acuta*	[50, 90]
		S. jponica var. *acuminata*	[17]
**40**	spiramine B	*S. japonica* var. *acuminata*	[11] [17]
		S. japonica var. *ovalifolia*	[47]
		S. japonica var. *glabra*	[94]
		S. japonica var. *insica*	[95]
		S. japonica var. *stellaris*	[25]
		S. japonica var. *acuta*	[90]
**41**	spiramine C2	*S. japonica* var. *acuminate*	[10]

**42**	spiramine U	*S. japonica* var. *acuta*	[90]
		S. japonica var. *acuta*	[96]
**43**	spiramine Q	*S. japonica* var. *insica*	[95, 97]
		S. japonica var. *stellaris*	[25]
		S. japonica var. *acuta*	[90, 96]
**44**	thalicsiline	*T. sessile*	[98]
**45**	spiramine T	*S. japonica* var. *acuta*	[90]
		S. japonica var. *acuta*	[96]
**46**	spiramine W	*S. japonica* var. *acuta*	[49]
**47**	spiramine P	*S. japonica* var. *insica*	[95]
		S. japonica var. *stellaris*	[25]
		S. japonica var. *acuta*	[49, 90, 96]
		S. japonica var. *acuminate*	[48]
**48**	19-oxodihydroatisine	*D. staphisagria*	[9]
**49**	22-*O*-acetyl-19-oxodihydroatisine	*D. staphisagria*	[9]
**50**	15,22-*O*-diacetyl-19-oxodihydroatisine	*D. staphisagria*	[9]
**51**	spiramine S	*S. japonica* var. *ovalifolia*	[89]
**52**	deacetylspiramine S/brunodelphinine A	*S. japonica* var. *ovalifolia*	[89]
		D. brunonianum	[99]
**53**	spiramine V	*S. japonica*	[32, 33]
**54**	spiramide	*S. japonica* var. *acuta*	[31]
**55**	spiramilactam A	*S. japonica* var. *ovalifolia*	[100]
**56**	spiramine R	*S. japonica* var. *insica*	[95]
**57**	spiramine X	*S. japonica* var. *acuta*	[93]
**58**	spiramine Y	*S. japonica* var. *acuta*	[93]
**59**	spiramilactams B	*S. japonica* var. *ovalifolia*	[100]
**60**	coryphidine	*A. coreanum*	[101]
**61**	azitine	*A. zeravschanicum*	[102]
		C. hellespontica	[7]
		C. raveyi	[28]
		D. staphisagria	[8, 9]
**62**	forrestline F	*D. forrestii* var. *viride*	[39]
**63**	spirimine A	*S. japonica* var. *acuminate*	[10]
**64**	19-*O*-deetylspiramine N	*S. japonica var. ovalifolia*	[103]
**65**	spiratine B	*S. japonica* var. *acuta*	[31]
		S. japonica var. *acuminate*	[48]
**66**	spiramine O	*S. japonica* var. *acuminata*	[104]
**67**	spirimine B	*S. japonica* var. *acuminate*	[10]
**68**	spiramine Z	*S. japonica* var. *acuta*	[93]
**69**	spiramine N	*S. japonica* var. *acuminata*	[105]
**70**	brunonine	*D. brunonianum*	[41]
**71**	spiramine J	*S. japonica* var. *acuminata*	[106]
**72**	spiramine L	*S. japonica* var. *acuminata*	[106]
**73**	spiramine M	*S. japonica* var. *acuminata*	[106]
**74**	spiramine K	*S. japonica* var. *acuminata*	[106]
**75**	13-(2-methylbutyryl)azitine	*D. scabriflorum*	[107]
**76**	isoazitine	*D. staphisagria*	[8]
**77**	leucostomine A	*A. leucostomum*	[108]
**78**	leucostomine B	*A. leucostomum*	[108]
**79**	barpubesine A	*A. barbatum var. puberulum*	[2]

**80**	barpubesine B	*A. barbatum var. puberulum*	[2]
**81**	uncinatine	*D. uncinatum*	[109]
**82**	aconicatisulfonine A	*A. carmichaelii*	[110]
**83**	aconicatisulfonine B	*A. carmichaelii*	[110]
**84**	barpuberudine	*A. barbatum* var. *puberulum*	[2]
**85**	brunonianine A	*D. brunonianum*	[111]
**86**	brunonianine B	*D. brunonianum*	[111]
**87**	brunonianine C	*D. brunonianum*	[111]
Atisine-type *Bis*-diterpenoid alkaloids			
**88**	bulleyanine B	*A. bulleyanum*	[56]
**89**	piepunine	*A. piepunense*	[112]
**90**	bulleyanine A	*A. bulleyanum*	[56]
**91**	staphisagrine	*D. staphisagria*	[113]
**92**	staphisagnine	*D. staphisagria*	[113]
**93**	staphigine	*D. staphisagria*	[114]
**94**	staphinine	*D. staphisagria*	[115]
**95**	staphidine	*D. staphisagria*	[115]
**96**	staphimine	*D. staphisagria*	[116]
**97**	staphirine	*D. staphisagria*	[116]
**98**	staphisine	*D. staphisagria*	[116]

Ref.

[1] U. Kolak, A. Turkekul, F. Ozgokce, A. Ulubelen, Two new diterpenoid alkaloids from Aconitum cochleare, Pharmazie 60(12) (2005) 953-5.

[2] N. Ablajan, B. Zhao, J.Y. Zhao, B.L. Wang, S.S. Sagdullaev, H.A. Aisa, Diterpenoid alkaloids from Aconitum barbatum var. puberulum Ledeb, Phytochemistry 181 (2021) 112567.

[3] B.T. Salimov, Z.K. Kuzibaeva, F.N. Dzhakhangirov, Structure-activity relationships of a series of alkaloids from Aconitum zeravschanicum and their analogs, Khim. Prir. Soedin. (3) (1996) 384-387.

[4] M. Nisar, M. Ahmad, N. Wadood, M.A. Lodhi, F. Shaheen, M.I. Choudhary, New diterpenoid alkaloids from Aconitum heterophyllum Wall: Selective butyrylcholinestrase inhibitors, J. Enzyme Inhib. Med. Chem. 24(1) (2009) 47-51.

[5] B.-N. Xing, S.-S. Jin, H. Wang, Q.-F. Tang, J.-H. Liu, R.-Y. Li, J.-Y. Liang, Y.-Q. Tang, C.-H. Yang, New diterpenoid alkaloids from Aconitum coreanum and their anti-arrhythmic effects on cardiac sodium current, Fitoterapia 94 (2014) 120-126.

[6] L.-h. Yang, C. Li, L.-m. Lin, Z.-m. Wang, Z. Li, Fat-soluble alkaloids from Aconitum tanguticum, Zhongguo Shiyan Fangjixue Zazhi 22(10) (2016) 32-36.

[7] H.K. Desai, B.S. Joshi, S.W. Pelletier, B. Sener, F. Bingol, T. Baykal, New alkaloids from Consolida hellespontica, Heterocycles 36(5) (1993) 1081-9.

[8] J.G. Diaz, J.G. Ruiz, G. De la Fuente, Alkaloids from Delphinium staphisagria, J. Nat. Prod. 63(8) (2000) 1136-1139.

[9] J.G. Díaz, J.G. Ruiz, G. de La Fuente, Alkaloids from Delphinium staphisagria, Journal of natural products 63(8) (2000) 1136-9.

[10] Y. Ma, Y.-M. Fan, X.-Y. Mao, L.-J. Huang, W. Gu, C. Yan, T. Huang, J.-X. Zhang, C.-M. Yuan, X.-J. Hao, Diterpene alkaloids and diterpenes from Spiraea japonica and their anti-tobacco mosaic virus activity, Fitoterapia 109 (2016) 8-13.

[11] X. Hao, M. Node, T. Taga, Y. Miwa, J. Zhou, S. Chen, K. Fuji, The structures of four new diterpene alkaloids, spiramines A, B, C, and D, Chem. Pharm. Bull. 35(4) (1987) 1670-2.

[12] S.W. Pelletier, N.V. Mody, An unusual rearrangement of ajaconine: an example of a "disfavored" 5-endo-trigonal ring closure, J. Am. Chem. Soc. 101(2) (1979) 492-4.
[13] S.W. Pelletier, The diterpene alkaloids. The structure of atidine, J. Am. Chem. Soc. 87(4) (1965) 799-802.
[14] S.W. Pelletier, R.S. Sawhney, H.K. Desai, N.V. Mody, The diterpenoid alkaloids of Consolida ambigua, J. Nat. Prod. 43(3) (1980) 395-406.
[15] Hajdu, P. Forgo, B. Loeffler, J. Hohmann, Diterpene and norditerpene alkaloids from Consolida orientalis, Biochem. Syst. Ecol. 33(10) (2005) 1081-1085.
[16] D. Dvornik, O.E. Edwards, Structure of ajaconine, Tetrahedron 14 (1961) 54-75.
[17] M. Node, X. Hao, J. Zhou, S. Chen, T. Taga, Y. Miwa, K. Fuji, Spiramines A, B, C, and D, new diterpene alkaloids from Spiraea japonica var. acuminata Franch, Heterocycles 30(1, Spec. Issue) (1990) 635-43.
[18] S. Huang, J.-F. Zhang, L. Chen, F. Gao, X.-L. Zhou, Diterpenoid alkaloids from Aconitum anthoroideum that offer protection against MPP+-Induced apoptosis of SH-SY5Y cells and acetylcholinesterase inhibitory activity, Phytochemistry (Elsevier) 178 (2020) 112459.
[19] H. Ahmad, S. Ahmad, S.A. Ali Shah, A. Latif, M. Ali, F. Ali Khan, M.N. Tahir, F. Shaheen, A. Wadood, M. Ahmad, Antioxidant and anticholinesterase potential of diterpenoid alkaloids from Aconitum heterophyllum, Bioorg. Med. Chem. 25(13) (2017) 3368-3376.
[20] J.-F. Zhang, Y. Li, F. Gao, L.-H. Shan, X.-L. Zhou, Four new C20-diterpenoid alkaloids from Aconitum rotundifolium, J. Asian Nat. Prod. Res. 21(7) (2019) 716-724.
[21] A.M. Nigmatullayaev, V.T. Salimov, Method of isolation and separation of individual alkaloids from the above-ground part of Aconitum zeravschanicum Steinb, Rastit. Resur. 36(4) (2000) 118-121.
[22] Z.-B. Li, F.-P. Wang, Two new diterpenoid alkaloids, beiwusines A and B, from Aconitum kusnezoffii, J. Asian Nat. Prod. Res. 1(2) (1998) 87-92.
[23] X. Hao, J. Zhou, S. Chen, K. Fuji, M. Node, New diterpene alkaloids from Spiraea japonica var. acuminata, Yunnan Zhiwu Yanjiu 13(4) (1991) 452-4.
[24] X. Hao, M. Node, J. Zhou, S. Chen, T. Taga, Y. Miwa, K. Fuji, Structures of spiramines E, F and G; the new diterpene alkaloids from Spiraea japonica var. acuminata Franch, Heterocycles 36(4) (1993) 825-31.
[25] J. Nie, X. Hao, Spiramilactone B, a new diterpenoid from Spiraea japonica var. stellaris, Yunnan Zhiwu Yanjiu 18(2) (1996) 226-228.
[26] F. Mericli, A.H. Mericli, A. Ulubelen, H.K. Desai, S.W. Pelletier, Norditerpenoid and Diterpenoid Alkaloids from Turkish Consolida orientalis, J. Nat. Prod. 64(6) (2001) 787-789.
[27] S. Dall'Acqua, B.B. Shrestha, M.B. Gewali, P.K. Jha, M. Carrara, G. Innocenti, Diterpenoid alkaloids and phenol glycosides from Aconitum naviculare (Bruhl) Stapf, Nat. Prod. Commun. 3(12) (2008) 1985-1989.
[28] A.H. Mericli, F. Mericli, V. Seyhan, A. Ulubelen, H.K. Desai, B.S. Joshi, Q. Teng, S.W. Pelletier, Isolation and structure of raveyine, a novel norditerpenoid alkaloid from Consolida raveyi (Boiss) Schrod, Heterocycles 45(10) (1997) 1955-1965.
[29] A. Ulubelen, A.H. Mericli, F. Mericli, U. Kolak, H.K. Desai, S.W. Pelletier, Diterpenoid alkaloids from the aerial parts of Delphinium staphisagria, Sci. Pharm. 67(3) (1999) 181-184.
[30] F. Mericli, H. Soydan, A.H. Mericli, Alkaloids from the aerial parts of a yellowish-brown flowering Delphinium peregrinum L. sample, Acta Pharm. Sci. 51(3) (2009) 219-223.
[31] H.-P. He, Y.-M. Shen, J.-X. Zhang, G.-Y. Zuo, X.-J. Hao, New diterpene alkaloids from the roots of

Spiraea japonica, J. Nat. Prod. 64(3) (2001) 379-380.
[32] G. De La Fuente Martin, L.R. Mesia, Stenocarpine, a diterpenoid alkaloid from Aconitella stenocarpa, Phytochemistry 46(6) (1997) 1087-1090.
[33] G.D.L.P. Martin, I. Ruiz Mesia, Secondary metabolites of Consolida aconiti, Bol. Soc. Quim. Peru 63(1) (1997) 44-49.
[34] G.D.L. Fuente, L. Ruiz-Mesia, J. Molero, C. Blanche, Diterpenoid alkaloids from Consolida axilliflora, Fitoterapia 67(1) (1996) 87-88.
[35] A. Ulubelen, A.H. Mericli, F. Mericli, H. Ozcelik, B. Sener, H. Becker, J. Zapp, I. Choudhary, R. Atta Ur, Norditerpene and diterpene alkaloids from Consolida hohenackeri, Phytochemistry 50(5) (1999) 909-912.
[36] A. Ulubelen, H.K. Desai, B.P. Hart, B.S. Joshi, S.W. Pelletier, A.H. Meriçli, H.Ç. Özen, Diterpenoid alkaloids from *Consolida oliveriana*, Journal of natural products 59(9) (1996) 907-910.
[37] A. Alva, M. Grandez, A. Madinaveitia, G. De La Fuente, J. Gavin, Three new norditerpenoid alkaloids from Consolida orientalis, Chem. Pharm. Bull. 52(5) (2004) 530-534.
[38] J.A. Goodson, Delphinium alkaloids. IV. Alkaloids of the seed of Delphinium ajacis, J. Chem. Soc. (1945) 245-6.
[39] S.W. Pelletier, N.V. Mody, R.C. Desai, Delcaroline, a novel alkaloid from Delphinium carolinianum Walt, Heterocycles 16(5) (1981) 747-50.
[40] S. Ahmad, H. Ahmad, H. Ullah Khan, A. Shahzad, E. Khan, S.A. Ali Shah, M. Ali, A. Wadud, M. Ghufran, H. Naz, M. Ahmad, Crystal structure, phytochemical study and enzyme inhibition activity of Ajaconine and Delectinine, J. Mol. Struct. 1123 (2016) 441-448.
[41] W. Deng, W.L. Sung, Brunonine: a new C20-diterpenoid alkaloid, Heterocycles 24(4) (1986) 869-72.
[42] S.W. Pelletier, F.M. Harraz, M.M. Badawi, S. Tantiraksachai, F. Wang, S. Chen, The diterpenoid alkaloids of Delphinium delavayi Franch var. pogonanthum (H.-M.) Wang, Heterocycles 24(7) (1986) 1853-65.
[43] S.W. Pelletier, S.A. Ross, H.K.J.P. Desai, A norditerpenoid alkaloid from Delphinium elatum, 29(7) (1990) 2381-2383.
[44] S.W. Pelletier, J.A. Glinski, B.S. Joshi, S. Chen, The diterpenoid alkaloids of Delphinium tatsienense Franch, Heterocycles 20(7) (1983) 1347-54.
[45] S.W. Pelletier, N.V. Mody, A.P. Venkov, S.B. Jones, Jr., Alkaloids of Delphinium virescens Nutt.: virescenine and 14-acetylvirescenine, Heterocycles 12(6) (1979) 779-82.
[46] S.D. Sastry, G.R. Waller, Mass spectral studies of the diterpenoid alkaloid, ajaconine, from Delphinium ajacis, Chem. Ind. (London) (9) (1972) 381-2.
[47] G.Y. Zuo, H.P. He, X. Hong, W.M. Zhu, X.S. Yang, X.J. Hao, New spiramines from Spiraea japonica var. ovalifolia, Heterocycles 55(3) (2001) 487-493.
[48] Y. Ma, X.-Y. Mao, L.-J. Huang, Y.-M. Fan, W. Gu, C. Yan, T. Huang, J.-X. Zhang, C.-M. Yuan, X.-J. Hao, Diterpene alkaloids and diterpenes from Spiraea japonica and their anti-tobacco mosaic virus activity, Fitoterapia 109 (2016) 8-13.
[49] B. Wang, B. Liu, G. Zuo, X. Hao, New minor diterpenoid alkaloid from Spiraea japonica var. acuta, Yunnan Zhiwu Yanjiu 22(2) (2000) 209-213.
[50] L. Li, Y.-M. Shen, X.-S. Yang, G.-Y. Zuo, Z.-Q. Shen, Z.-H. Chen, X.-J. Hao, Antiplatelet aggregation activity of diterpene alkaloids from Spiraea japonica, Eur. J. Pharmacol. 449(1-2) (2002) 23-28.
[51] L.-H. Hou, D.-L. Chen, X.-X. Jian, F.-P. Wang, Three new diterpenoid alkaloids from roots of

Aconitum ouvrardianum Hand-Mazz, Chem. Pharm. Bull. 55(7) (2007) 1090-1092.
[52] A.H. Mericli, F. Mericli, A. Ulubelen, M. Bahar, R. Ilarslan, G. Algul, H.K. Desai, Q. Teng, S.W. Pelletier, Diterpenoid alkaloids from the aerial parts of *Aconitum anthora* L, Pharmazie 55(9) (2000) 696-698.
[53] S. Huang, J.F. Zhang, L. Chen, F. Gao, X.L.J.P. Zhou, Diterpenoid alkaloids from Aconitum anthoroideum that offer protection against MPP+–Induced apoptosis of SH–SY5Y cells and acetylcholinesterase inhibitory activity, 178 (2020) 112459.
[54] D. Chen, W. Song, Alkaloids of tangut monkshood (Aconitum tanguticum), Zhongcaoyao 16(8) (1985) 338-42.
[55] L.-h. Yang, C. Li, L.-m. Lin, Z.-m. Wang, Z. Li, Chemical constituents from whole plants of Aconitum tanguticum (IV), Zhongguo Shiyan Fangjixue Zazhi 22(4) (2016) 39-44.
[56] X.-Y. Duan, D.-K. Zhao, Y. Shen, Two new bis-C20-diterpenoid alkaloids with anti-inflammation activity from Aconitum bulleyanum, J. Asian Nat. Prod. Res. 21(4) (2019) 323-330.
[57] X. Wang, X. Shu, X. Wang, J. Yu, F. Jing, Preparative isolation of seven diterpenoid alkaloids from Aconitum coreanum by pH-zone-refining counter-current chromatography, Molecules 19(8) (2014) 12619-12629, 11 pp.
[58] S. Sakai, N. Shinma, S. Hasegawa, T. Okamoto, On the alkaloids of Aconitum gigas Lev. et Van. and the structure of a new base, gigactonine, Yakugaku Zasshi 98(10) (1978) 1376-84.
[59] S. Jiang, S. Guo, B. Zhou, S. Wang, F. Yi, L. Ji, Alkaloids from Aconitum gymnandrum Maxim (I), Yaoxue Xuebao 21(4) (1986) 279-84.
[60] S.W. Pelletier, N.V. Mody, J. Finer-Moore, H.K. Desai, H.S. Puri, The structure and absolute configuration of heterophylloidine, Tetrahedron Lett. 22(4) (1981) 313-14.
[61] S.W. Pelletier, R. Aneja, K.W. Gopinath, Alkaloids of Aconitum heterophyllum: isolation and characterization, Phytochemistry 7(4) (1968) 625-35.
[62] O.E. Edwards, T. Singh, Atisine: the heterocyclic ring and functional groups, Can. J. Chem. 32 (1954) 465-73.
[63] W.R. Dunstan, T.A. Henry, Contributions to our knowlegde of the aconite alkaloids. Part XVIII. The aconitine group of alkaloids, J. Chem. Soc., Trans. 87 (1905) 1650-1656.
[64] L. Gao, X. Wei, L. Yang, A new diterpenoid alkaloid from a Tibetan medicinal herb Aconitum naviculare Stapf, J. Chem. Res. (4) (2004) 307-308.
[65] P. Wangchuk, J.B. Bremner, S. Samosorn, Hetisine-Type Diterpenoid Alkaloids from the Bhutanese Medicinal Plant Aconitum orochryseum, J. Nat. Prod. 70(11) (2007) 1808-1811.
[66] Q. Jiang, S.W. Pelletier, Two New Diterpenoid Alkaloids from Aconitum palmatum, J. Nat. Prod. 54(2) (1991) 525-531.
[67] C. Peng, J. Wang, X. Jian, F. Wang, Alkaloids of Aconitum sinomontanum and Aconitum racemulosum Franch var. pengzhouense, Tianran Chanwu Yanjiu Yu Kaifa 12(4) (2000) 45-51.
[68] E.G. Zinurova, T.V. Khakimova, L.V. Spirikhin, M.S. Yunusov, Alkaloids of Aconitum septentrionale seeds, Chem. Nat. Compd. 36(4) (2001) 387-389.
[69] Y. He, Z. Ma, Q. Yang, B. Yao, L. Gao, A new norditerpenoid alkaloid from Aconitum taipaicum, Yaoxue Xuebao 43(9) (2008) 934-937.
[70] H. Wang, S. Jiang, P. Yang, M. Ying, S. Lin, D. Zhu, Alkaloids from Aconitum Tanguticum, Tianran Chanwu Yanjiu Yu Kaifa 14(4) (2002) 13-15.
[71] J. Li, C. Hang, W. Zhao, H. Pi, H. Ruan, Z.J.H.C.A. Peng, New Alkaloids from Aconitum vaginatum, 97(5) (2014).

[72] F. Mericli, A.H. Mericli, H.K. Desai, A. Ulubelen, S.W. Pelletier, Diterpenoid alkaloids from Consolida regalis S.F. Gray subsp. paniculata (host) Soo var. paniculata, Sci. Pharm. 69(1) (2001) 63-67.
[73] Y.-Q. He, Z.-Y. Ma, Q. Yang, B.-Z. Du, Z.-X. Jing, B.-H. Yao, M.T. Hamann, Diterpenoid alkaloids and flavonoids from Delphinium albocoeruleum Maxim, Biochem. Syst. Ecol. 38(4) (2010) 554-556.
[74] Y.Q. He, Z.Y. Ma, X.M. Wei, D.J. Liu, B.Z. Du, B.H. Yao, L.M. Gao, Honatisine, a Novel Diterpenoid Alkaloid, and Six Known Alkaloids from Delphinium honanense and Their Cytotoxic Activity, Chem. Biodiversity 8(11) (2011) 2104-2109.
[75] G. de la Fuente, L. Ruiz-Mesia, Norditerpenoid alkaloids from Delphinium peregrinum var. elongatum, Phytochemistry 39(6) (1995) 1459-65.
[76] L.C. Craig, W.A. Jacobs, Aconite alkaloids. XIV. Oxidation of the hydrocarbon from the dehydrogenation of atisine, J. Biol. Chem. 152 (1944) 651-7.
[77] S.W. Pelletier, N.V. Mody, The conformational analysis of the E and F rings of atisine, veatchine, and related alkaloids. The existence of C-20 epimers, J. Am. Chem. Soc. 99(1) (1977) 284-6.
[78] Y.Q. He, X.M. Wei, Y.L. Han, L.M. Gao, Two new diterpene alkaloids from Delphinium chrysotrichum, Chin. Chem. Lett. 18(5) (2007) 545-547.
[79] Y.-Q. He, Z.-Y. Ma, X.-M. Wei, B.-Z. Du, Z.-X. Jing, B.-H. Yao, L.-M. Gao, Chemical constituents from Delphinium chrysotrichum and their biological activity, Fitoterapia 81(7) (2010) 929-931.
[80] G.Y. Zuo, H.P. He, X. Hong, W.M. Zhu, X.S. Yang, X.J. Hao, New spiramines from *Spiraea japonica* var. *ovalifolia*, Heterocycles 55(3) (2001),) 487-493.
[81] F.N. Dzhakhangirov, I.A. Bessonova, Alkaloids of Aconitum coreanum. X. Curare-like activity-structure relationship, Chem. Nat. Compd. 38(1) 74-77.
[82] Y. Sun, Y. Sun, Chemical constituents of Aconitum coreanum (Levl.), Zhongguo Zhongyao Zazhi 16(12) (1991) 738-9.
[83] M.G. Reinecke, W.H. Watson, D.C. Chen, W.M. Yan, The case of the troubling doubling. Isoatisine and 19-epiisoatisine from the Chinese herb guan-bai-fu (Aconitum koreanum), J. Org. Chem. 52(22) (1987) 5051-3.
[84] Q. Jiang, S.W. Pelletier, Four new diterpenoid alkaloids from Aconitum palmatum Don, Tetrahedron Lett. 29(16) (1988) 1875-8.
[85] Y.-Q. He, B.-H. Yao, Z.-Y. Ma, Diterpenoid alkaloids from a Tibetan medicinal plant Aconitum richardsonianum var. pseudosessili florum and their cytotoxic activity, J. Pharm. Anal. 1(1) (2011) 57-59.
[86] D.M. Razakova, I.A. Bessonova, M.S. Yunusov, Atisine chloride and isoatisine from Aconitum coreanum and A. rotundifolium, Khim. Prir. Soedin. (2) (1988) 309-10.
[87] Z.M. Vaisov, B.T. Salimov, B. Tashkhodzhaev, M.S. Yunusov, Alkaloids of Aconitum zeravschanicum, Khim. Prir. Soedin. (5) (1986) 658-9.
[88] Y. He, Z. Ma, Q. Yang, X. Yu, L. Gao, B. Yao, Study on chemical constituents from Delphinium honanense var. piliferum, Zhongguo Zhongyao Zazhi 33(23) (2008) 2784-2786.
[89] G.Y. Zuo, H.P. He, X. Hong, W.M. Zhu, Y.M. Hu, X.S. Yang, X.J. Hao, New diterpenoid alkaloids from Spiraea japonica var. ovalifolia, Chin. Chem. Lett. 12(2) (2001) 147-150.
[90] J. Nie, X. Hao, Diterpene alkaloids from Spiraea japonica var. acuta, Yunnan Zhiwu Yanjiu 19(4) (1997) 429-432.
[91] Y. He, D. Zhang, L.M. West, Delphatisine D and Chrysotrichumine A, two new diterpenoid alkaloids from Delphinium chrysotrichum, Fitoterapia 139 (2019) 104407.
[92] M.H. Toda, Yoshimasa, Structures of spiradines F and G from Spiraea japonica, Tetrahedron

Letters
53 (1968) 5565-5568.
[93] B.-G. Wang, L. Li, X.-S. Yang, Z.-H. Chen, X.-J. Hao, Three new diterpene alkaloids from Spiraea japonica, Heterocycles 53(6) (2000) 1343-1350.
[94] X. Yang, X. Hao, The diterpenoid alkaloids from Spiraea japonica var. glabra, Yunnan Zhiwu Yanjiu 15(4) (1993) 421-3.
[95] X.-J. Hao, X. Hong, X.-S. Yang, B.-T. Zhao, Diterpene alkaloids from roots of Spiraea Japonica, Phytochemistry 38(2) (1995) 545-7.
[96] B.-G. Wang, X. Hong, G.-Y. Zuo, X.-J. Hao, Structural revision of four spiramine diterpenoid alkaloids from the roots of Spiraea japonica, J. Asian Nat. Prod. Res. 2(4) (2000) 271-281.
[97] Z. Shen, Z. Chen, L. Li, W. Lei, X. Hao, Antiplatelet and antithrombotic effects of the diterpene spiramine Q from Spiraea japonica var. incisa, Planta Med. 66(3) (2000) 287-289.
[98] Y.C. Wu, T.S. Wu, M. Niwa, S.T. Lu, Y. Hirata, Alkaloids of Formosan Thalictrum sessile, Phytochemistry 27(12) (1988) 3949-53.
[99] C. Lin, C. Zhu, Y. Yao, Y. Yuan, C20-diterpene alkaloid compound extracted from Delphinium brunonianum, its preparation method and application in preparing drug for preventing and treating non-alcoholic fatty liver, Guangzhou University of Chinese Medicine Guangzhou Academy of Chinese Medicine , Peop. Rep. China . 2021, p. 29pp.
[100] H.-Y. Liu, W. Ni, C.-X. Chen, Y.-T. Di, X.-J. Hao, Two New Diterpenoid Lactams from Spiraea japonica var. ovalifolia, Helv. Chim. Acta 92(6) (2009) 1198-1202.
[101] I.A. Bessonova, M.R. Yagudaev, M.S. Yunusov, Alkaloids of Aconitum coreanum. VIII. Structure of coryphidine, Khim. Prir. Soedin. (2) (1992) 243-246.
[102] B.T. Salimov, Atidine and atisine azomethine from Aconitum zeravschanicum, Khim. Prir. Soedin. (1) (1993) 84-5.
[103] G. Zuo, H. He, X. Hong, W. Zhu, X. Yang, X.J.H. Hao, New spiramines from Spiraea japonica var. ovalifolia, 55(3) (2001) 487.
[104] X. Hao, M. Node, J. Zhou, S. Chen, K. Fuji, Chemical structures of spiramine H, I and O, Yunnan Zhiwu Yanjiu 16(3) (1994) 301-4.
[105] X. Hao, J. Zhou, K. Fuji, M. Node, The chemical structures of spiramine N and spiraminol, Chin. Chem. Lett. 3(6) (1992) 427-30.
[106] X. Hao, J. Zhou, K. Fuji, M. Node, The chemical structures of spiramine J, K, L, and M, Yunnan Zhiwu Yanjiu 14(3) (1992) 314-18.
[107] Diterpenoid Alkaloids from the Roots of Delphinium scabriflorum, (2004).
[108] W. Xu, L. Chen, L. Shan, F. Gao, S. Huang, X. Zhou, Two new atisine-type C20-diterpenoid alkaloids from Aconitum leucostomum, Heterocycles 92(11) (2016) 2059-2065.
[109] A. Ulubelen, M. Arfan, U. Sonmez, A.H. Mericli, F. Mericli, Diterpenoid alkaloids from Delphinium uncinatum, Phytochemistry 47(6) (1998) 1141-1144.
[110] Y. Wu, S. Shao, Q. Guo, C. Xu, H. Xia, T. Zhang, J. Shi, Aconicatisulfonines A and B, analgesic zwitterionic C20-diterpenoid alkaloids with a rearranged atisane skeleton from Aconitum carmichaelii, Org. Lett. 21(17) (2019) 6850-6854.
[111] Q. Li, Z.W. Wang, M.X. Wang, H.L. Yu, L. Chen, Z. Cai, Y. Zhang, M.M. Gu, Y.L. Shao, H.P. Han, Z.X. Liao, Brunonianines A-C, C_{20}-diterpenoid alkaloids with cyano group from *Delphinium brunonianum* Royle, Phytochemistry 219 (2024) 113987.
[112] L. Cai, L. Song, Q.-H. Chen, X.-Y. Liu, F.-P. Wang, Piepunine, a novel bis-diterpenoid alkaloid from

the roots of Aconitum piepunense, Helv. Chim. Acta 93(11) (2010) 2251-2255.
[113] S.W. Pelletier, Z. Djarmati, N.V. Mody, The structures of staphisagnine and staphisagrine, two novel bis-diterpene alkaloids from Delphinium staphisagria, Tetrahedron Lett. (21) (1976) 1749-52.
[114] S.W. Pelletier, N.V. Mody, Z. Djarmati, S.D. Lajsic, The structures of staphigine and staphirine. Two novel bisditerpene alkaloids from Delphinium staphisagria, J. Org. Chem. 41(18) (1976) 3042-4.
[115] S.W. Pelletier, N.V. Mody, Z. Djarmati, I.V. Micovic, J.K. Thakkar, The structures of staphidine, staphinine, and staphimine, three novel bis-diterpene alkaloids from Delphinium staphisagria, Tetrahedron Lett. (14) (1976) 1055-8.
[116] S.W. Pelletier, N.V. Mody, Z. Djarmati, S.D. Lajsic, The structures of staphigine and staphirine. Two novel bisditerpene alkaloids from Delphinium staphisagria, J. Org. Chem. 41(18) (1976) 3042-3044.
